# Isotopic Fractionation and Kinetic Isotope Effects of a Purified Bacterial Nitric Oxide Reductase (NOR)

**DOI:** 10.1021/acs.biochem.5c00417

**Published:** 2025-10-10

**Authors:** Elise D. Rivett, Clarisse M. Finders, Joshua A. Haslun, Hasand Gandhi, Maximilian Kahle, Pia Ädelroth, Peggy H. Ostrom, Nathaniel E. Ostrom, Eric L. Hegg

**Affiliations:** † Department of Biochemistry & Molecular Biology, 3078 Michigan State University, East Lansing, Michigan 48824, United States; ‡ DOE Great Lakes Bioenergy Research Center, Michigan State University, East Lansing, Michigan 48824, United States; § Department of Integrative Biology, Michigan State University, East Lansing, Michigan 48824, United States; ∥ Department of Biochemistry and Biophysics, 7675 Stockholm University, Stockholm SE-106 91, Sweden

## Abstract

Nitrous oxide (N_2_O) is a serious concern due to its role in global warming and ozone destruction. Agricultural practices account for ∼80% of all anthropogenic N_2_O produced in the US, due in large part to the stimulation of microbial denitrification. Stable isotopes are uniquely suited to examine both microbial N_2_O sources and the mechanism of N_2_O biosynthesis through the use of Site Preference (δ^15^N^SP^; the difference in δ^15^N between the central and outer N atoms in N_2_O) and kinetic isotope effects (KIEs), respectively. Using trace gas isotope ratio mass spectrometry (TG-IRMS), we determined the δ^15^N, δ^15^N^α^, δ^15^N^β^, and δ^18^O of N_2_O produced by a purified cytochrome *c* nitric oxide reductase (cNOR) from *Paracoccus denitrificans*. We also calculated δ^15^N^SP^, the KIEs, and associated isotopic enrichment factors (ε) for N^bulk^, N^α^, and N^β^. A normal isotope effect was observed for bulk ^15^N, with a KIE value of 1.0086 ± 0.0009 (ε = −8.6 ± 0.9‰). The isotope effects for both ^15^N^α^ and ^15^N^β^ were also normal, with position-specific KIEs of 1.0072 ± 0.0010 (ε = −7.2 ± 1.0‰) and 1.0100 ± 0.0010 (ε = −9.9 ± 1.0‰), respectively, and δ^15^N^SP^ values ranged from 0.5 to 8.7‰ with no significant trend as the reaction proceeded. Values of δ^18^O increased with N_2_O production (slope of δ^18^O against [−*f* ln *f*/(1 – *f*)] = −19.9 ± 1.9‰). We present implications for the mechanism of N_2_O production from cNOR based on our data.

## Graphical abstract

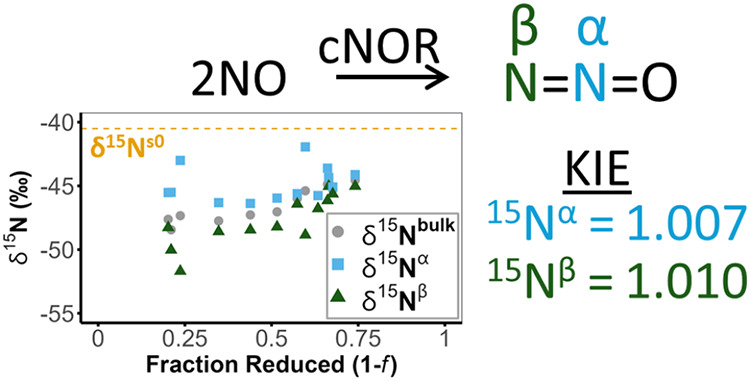

## Introduction

Nitrogen is a key element to all life and exists as either reactive nitrogen species or dinitrogen gas (N_2_).^1^ These two reservoirs are connected to each other through an intricate cycle of pathways that includes nitrification, denitrification, anammox, comammox, dissimilatory nitrate reduction to ammonia (DNRA), and potentially others.^1,2^

Nitrous oxide (N_2_O) is one of the key intermediates in the nitrogen cycle. Unfortunately, N_2_O is also a potent greenhouse gas, with a global warming potential (100-year time horizon) approximately 300 times greater than that of carbon dioxide (CO_2_),[Fn fn1] and N_2_O is also the largest anthropogenic source of ozone depletion.^3,5^ Since the invention of the Haber-Bosch process, which fixes N_2_ to ammonia, humans have had an enormous impact on both the amount and flux of bioavailable nitrogen, including N_2_O. Currently, anthropogenic N_2_O accounts for ∼40% of all N_2_O generated,^6^ and in the US, ∼79%[Fn fn2] of this N_2_O is produced by microbial activity in response to agricultural practices.^7^ With atmospheric N_2_O concentrations increasing by an average of ∼ 0.29% every year from 2001–2022,^8^ fully understanding the enzymatic processes that directly contribute to anthropogenic agricultural N_2_O production is especially important.

Soil microbial processes that produce N_2_O include nitrification, denitrification, and nitrifier-denitrification. Nitrification is the conversion of ammonia/ammonium (NH_3_/NH_4_^+^) to nitrate (NO_3_^–^) via hydroxylamine (NH_2_OH) under aerobic conditions. During the oxidation of NH_2_OH, N_2_O is released as a side product. Denitrification and nitrifier-denitrification are processes that under anaerobic conditions convert NO_3_^–^ and NO_2_^–^, respectively, to N_2_ through a series of intermediates including nitric oxide (NO) and N_2_O. Nitric oxide reductases (NOR), which produce N_2_O from NO, are one of the most important types of enzyme in soil microbial N_2_O production.^2^ NOR activity can be found in both bacteria and fungi.^2,9^ In its most generic form, NOR catalyzes the following reaction:

1 $$2\mathrm{NO}+2e^{-}+2H^{+}\to N_{2}O+H_{2}O$$

A single NOR consisting of one subunit (46 kDa) is present in fungi. A P450-type heme serves as the catalytic center of the enzyme, giving P450nor its name.^10^ Catalysis begins when an NO molecule binds to the ferric P450 heme, producing a heme-NO complex. The heme-NO complex is then reduced by hydride transfer from NAD­(P)­H, producing a singly protonated intermediate that is best described as (Fe­(III)-NHO^•–^).^11–14^ The details of the final steps of the reaction that include binding of the second NO molecule followed by N_2_O formation are less understood.^15^ It is also possible that flavohemoglobin may contribute to N_2_O production in some fungal denitrifiers.^16–18^

In contrast to fungi, bacterial enzymes involved in NO reduction to N_2_O include both soluble enzymes [e.g., flavodiiron proteins (FDPs),^19^ flavohemoglobin,^20^ and the hybrid cluster protein (Hcp)^21,22^ ] and members of the membrane-bound heme-copper oxidase superfamily^15,23,24^ [e.g., quinol-dependent NOR (qNOR),^25^ copper-containing NOR (Cu_A_NOR),^26^ and cytochrome *c*-dependent NOR (cNOR)^24,27^ ]. Of these, the best studied bacterial NOR is cNOR, which is composed of two subunits, NorC (∼17 kDa) and NorB (∼56 kDa). NorC contains one transmembrane helix and a cytochrome *c* domain that transfers electrons from the soluble donor (cytochrome *c* or pseudoazurin) to NorB. NorB is the catalytic unit and is an integral membrane protein with 12 transmembrane helices. Contained in the helices are two heme *b* molecules, termed heme *b* and heme *b*_3_, analogous to the cytochrome *c* oxidase nomenclature.^28^ Heme *b* receives electrons from NorC and passes them to heme *b*_3_, which makes up half of the binuclear catalytic site. The other half of this site is a nonheme iron, termed Fe_B_, coordinated to three histidines and a glutamate.^28^

Compared to our understanding of P450nor, our knowledge of the bacterial NOR mechanism is more limited. NO is known to inhibit cNOR at concentrations above ∼5 μM, but the mechanism by which substrate inhibition occurs is still under debate.^29–33^ Also unresolved is the exact site of NO binding during turnover, or how the N–N bond is formed during N_2_O production.

Three general classes of mechanisms can be envisioned for cNOR (Scheme 1), mainly differing in where the NO molecules bind to the catalytic center and how the N–N bond is formed. Two of the mechanisms propose that all of the chemistry occurs on one of the two active-site Fe centers, with the first NO molecule binding to either the nonheme Fe_B_ (*cis-*Fe_B_ mechanism) or the heme iron (*cis*-heme *b*_3_ mechanism) before being attacked by the second NO molecule.^34,35^ The third mechanism, known as the *trans* mechanism, suggests that one NO molecule binds to each iron prior to N–N bond formation.^29,36–38^ Each mechanism predicts formation of a hyponitrite anion with unique coordination to the active site Fe centers (Scheme 1). This hyponitrite intermediate must then release N_2_O via cleavage of an N–O bond. While decades of research on cNOR have revealed that NO can bind either Fe_B_ or heme *b*_3_,^31,36–39^ the hyponitrite intermediate has not been observed under normal reaction conditions,[Fn fn3] and it remains unclear which proposed mechanism is most accurate.

**Scheme 1:**
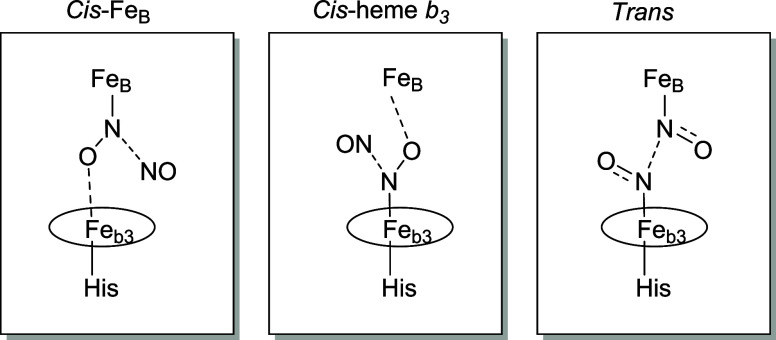
Three Potential Transition States during N_2_O Production by cNOR, Adapted from Blomberg^a^ ^35^

a The catalytic site is a binuclear center that consists of the heme *b*_3_ and a non-heme iron, Fe_B_.

Stable isotopes have a long history of being used to study enzymatic mechanisms through measuring kinetic isotope effects (KIEs).^42–45^ A KIE is defined as the rate constant of the light isotope divided by the rate constant of the heavy isotope. KIEs are useful in determining mechanisms and transition state (TS) structures because they provide a snapshot of the vibrational environment of the bonds of interest.^46^ The two nitrogen atoms in the N_2_O molecule are not equivalent, and therefore the center nitrogen (termed α) and the terminal nitrogen (termed β, Scheme 2) will each have their own KIE, making stable isotopes particularly well-suited to investigate N_2_O biosynthesis. In this context, we previously employed stable isotopes to examine the production of N_2_O by purified P450nor from *Histoplasma capsulatum*.^47^ More recently, we developed a new isotopic model, the Expanded Rayleigh model, which allows for significantly more accurate determination of *position-specific* KIEs for N^α^ and N^β^ than the model previously used (the Rayleigh distillation equation).^48^ In this manuscript, we present the δ^15^N, δ^15^N^α^, δ^15^N^β^, and δ^18^O values for N_2_O produced by purified cNOR from the bacterium *Paracoccus denitrificans*. We also present the bulk and position-specific isotopic enrichment factors and KIEs determined from δ^15^N, δ^15^N^α^, and δ^15^N^β^. Additionally, we discuss the different contributors to the KIE and the mechanistic implications of our data.

**Scheme 2:**
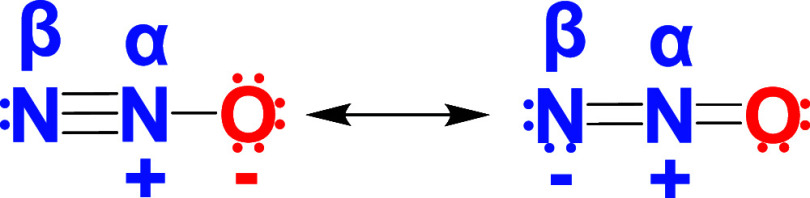
Resonance Structures of Nitrous Oxide Showing the Asymmetry of the N_2_O Molecule^a^

a The nitrogen atoms are in blue, and the oxygen atom is in red. N^β^ is the terminal nitrogen while N^α^ is the central atom bonded to both nitrogen and oxygen.

## Materials and Methods

NO was purified by attaching a stainless steel trap (20 cm × 0.6 cm) and a 160 mL side arm flask to a cylinder of 99.5% NO (Airgas) in a chemical fume hood. Molecular sieves (5A, 8 × 12 mesh; Arcos Organics) in both the trap and flask were activated by heating the system to 180 °C under vacuum (∼10^–2^ Torr) for 24 h. After cooling to room temperature, NO was allowed to enter the evacuated flask after passing through the molecular sieve trap.

All other reagents were purchased from Sigma-Aldrich unless otherwise noted and used without further purification. *P. denitrificans* cNOR (NorB, UniProtKB Q51663 (A1B4X6); NorC, UniProtKB Q51662 (A1B4X7)) was expressed in and purified from *Escherichia coli*, based on the method developed by Butland et al.,^49^ with the modifications described by Flock et al.^50^

### Enzymatic Assays

The cNOR activity assay (12.8 mL) was prepared by mixing 50 mM anaerobic 4-(2-hydroxyethyl)-1-piperazineethanesulfonic acid (HEPES) buffer (pH 7.5), 50 mM KCl, 0.5 mM *N*,*N*,*N*′,*N*′-tetramethyl-*p*-phenylenediamine (TMPD, Arcos Organics), 3 mM ascorbic acid, 20 μM equine heart cytochrome *c*, and 0.04% *n*-dodecyl-β-d-maltoside (DDM, Anatrace) in a 160 mL glass serum bottle in an anaerobic chamber (Coy). After the serum bottle was capped with a butyl rubber stopper (Geo-Microbial Technologies, Inc.) and crimped, NO gas (10.3 μmols) was injected into the headspace of the serum bottle (147.2 mL) using a gastight syringe (Hamilton) and allowed to equilibrate for 15 min while shaking at 100 rpm at room temperature (approximately 20–22 °C) on a benchtop shaker (Thermo Scientific). To avoid potential diffusion of N_2_O out of the reaction vial, the serum bottle was inverted before the cNOR (20 μL, 78 nM final concentration) was injected to initiate the reaction. The bottle was continuously shaken at 100 rpm during the course of the reaction. Prior to sampling, a volume of N_2_ gas equivalent to the intended sample was injected to maintain a constant pressure upon sampling. The gas sample was then injected into a 30 mL sealed serum bottle that had been sparged with ultrahigh purity N_2_. To ensure the enzyme was producing N_2_O at the expected rate, one replicate was analyzed via gas chromatography (GC) on a Shimadzu Greenhouse Gas Analyzer, model GC-2014 (Columbia, MD), equipped with a Haysep N separation column and an electron capture detector (ECD). The GC conditions used were: 5% CH_4_/95% Ar make up gas (Airgas) with a flow rate = 2.5 mL/min; N_2_ carrier gas with a flow rate = 25 mL/min; 100 °C GC oven temperature; 350 °C ECD temperature. To obtain the isotopic composition of the N_2_O produced, samples were analyzed on an IsoPrime 100 stable isotope ratio mass spectrometer (IRMS) interfaced to a TraceGas inlet system (Elementar; Mt. Laurel, NJ) as previously described.^47^ As the entire sample was required for IRMS analysis, the δ values for each sample were measured only once. The δ^15^N value was evaluated based on the *m*/*z* 45:44 ratio while the δ^15^N^α^ value was based on the *m*/*z* 31:30 ratio of the NO fragment ion generated in the mass spectrometer. The δ^15^N^β^ value was then calculated from the equation δ^15^N = (δ^15^N^α^ + δ^15^N^β^)/2 and corrected for mass overlap based on the method described by Toyoda and Yoshida.^51^ Sample reproducibility (1 standard deviation) based on N_2_O isotopic analysis of standards was 0.5, 0.5, 0.7, 0.7, and 1.2‰ for δ^15^N, δ^18^O, δ^15^N^α^, δ^15^N^β^, and δ^15^N^SP^, respectively. The δ^18^O, δ^15^N, δ^15^N^α^, and δ^15^N^β^ values of the laboratory pure N_2_O standard were 40.15, −0.72, 10.19, and −11.65‰, respectively, as determined by calibration against the secondary standards USGS51 and USGS52.^52^ The N_2_O concentrations were obtained by quantifying the peaks from the IRMS relative to a standard curve. For each time point, the total amount (nmol) of enzymatically produced N_2_O was calculated by adding the amount of N_2_O present in the reaction bottle at that time to the amount of N_2_O removed from the reaction bottle during previous sampling time points. NO concentrations were not measured directly due to the reactivity of NO, but they were calculated by subtracting 2*­(nmol N_2_O) from the initial amount of NO.

Two different sets of experiments were performed. The first set was performed to determine the KIEs observed during the production of N_2_O by cNOR. Three replicate reaction mixtures were prepared from the same enzyme purification, and the headspace was sampled every 40 min until enzymatic N_2_O production began to plateau (∼240 min). Data from these three reaction vials were pooled and analyzed as described below. To determine if isotopic exchange between the water and NO was occurring, a second set of experiments was performed. Four replicate reaction mixtures were prepared, two of which were spiked with ^18^O enriched water (87‰). Samples were taken every 30 min for 180 min (Figures S1–S4) until they spanned at least 10% reaction completion. Data from the spiked and nonspiked replicates were pooled separately. A set of control experiments to test for the abiotic production of N_2_O was also performed. Two replicate reaction mixtures were prepared on separate days. Each reaction mixture included 40.9 μmol NO (four times the amount used in the reactions with enzyme) and all other reagents except the cNOR enzyme. The headspace of one no-enzyme control reaction was sampled at 30 and 120 min; the headspace of the second no-enzyme control was sampled at 60 and 180 min. N_2_O above background was not detected via GC-ECD analysis in our control reactions (Figure 1), indicating that the N_2_O observed in the first two sets of experiments was enzymatically produced.

**Figure 1:**
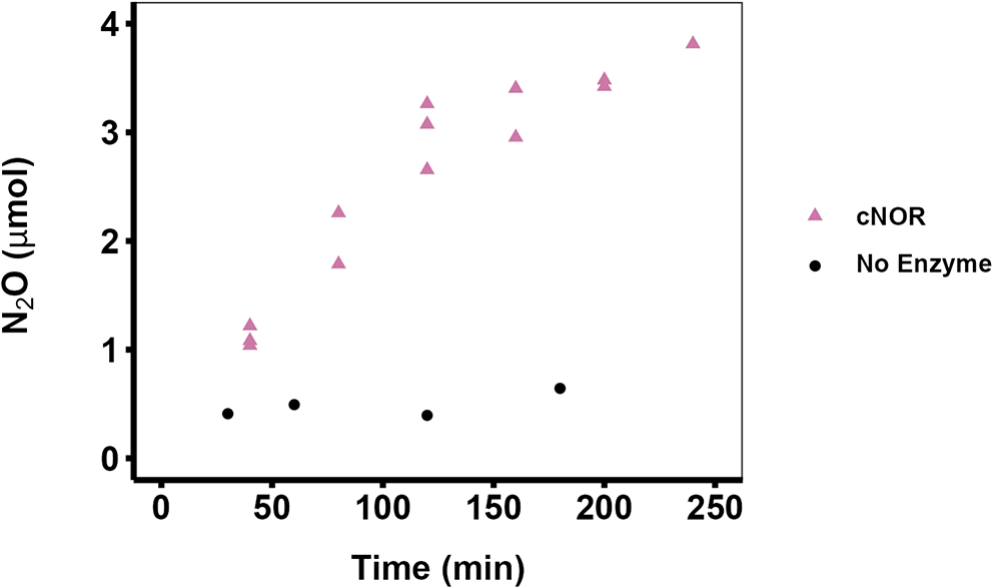
Production of N_2_O in the presence and absence of *P. denitrificans* cNOR. Reactions with purified cNOR (purple triangles): Three reaction mixtures were prepared on the same day, each containing 78 nM cNOR and 10.3 μmol of NO (5.15 μmol is the maximum amount of N_2_O that can be formed). N_2_O was quantified by IRMS. Note that there are two overlapping data points at 40 and 200 min. Reactions without cNOR (black circles): Two reaction mixtures were prepared (on two different days), each containing 40.9 μmol of NO gas (20.45 μmol is the maximum amount of N_2_O that can be formed). N_2_O was quantified by ECD. The concentrations of HEPES buffer (pH 7.5) and the other reagents were the same in both sets of experiments.

### Isotope Nomenclature

The isotopic ratio R is defined as the abundance of the heavy isotope over the abundance of the light isotope. In the context of this manuscript, which analyzes N_2_O, this is equivalent to the abundance of ^15^N and ^18^O over the abundance of ^14^N and ^16^O, respectively.

2 R=heavyisotopelightisotope=(e.g.,N15N14)

Isotope values are expressed using the delta (δ) notation.

3 $$\delta =\left(\left(\frac{R_{\mathrm{sample}}}{R_{\mathrm{standard}}}\right)-1\right)\times 1000$$

The international standards for δ^18^O and δ^15^N are Vienna Standard Mean Ocean Water and atmospheric N_2_, respectively.^53^ Mass overlap corrections (e.g., due to the presence of N_2_^17^O) were performed following the procedure of Toyoda and Yoshida.^51^

### KIE, ε, and δ^15^N^SP^

For a closed system with a single, irreversible step or an irreversible multistep reaction with a single rate-limiting step, isotopic fractionation can be modeled using Mariotti’s approximation of the Rayleigh equation^54^

4 δN15p=δN15s0−εp/s(f×ln⁡f1−f)

where δ^15^N^p^ is the δ^15^N value of the product, *f* is the fraction of the substrate remaining, and the slope, ε_p/s_, is the isotopic enrichment factor (a measure of the isotopic discrimination or the relative reaction rates of the heavy and light isotopes). The intercept of this equation, δ^15^N^s0^, is the initial δ value of the substrate. This approximation of the Rayleigh equation, which is written with the product δ value as the dependent variable, introduces systematic errors which are typically smaller than the analytical error associated with δ value measurements.^48,55^

In the case of N_2_O biosynthesis, eq 4 is only used to determine the enrichment factor for bulk N (ε_N‑bulk_) and the δ^15^N^s0^ value for NO because this equation is only accurate for bulk N (i.e., the average of δ^15^N^α^ and δ^15^N^β^ in N_2_O).^48^ Thus, δ^15^N^p^ refers to bulk δ^15^N, and ε_*p*/*s*_ refers to the bulk enrichment factor, ε_N‑bulk_. The fraction of NO remaining (*f*) was calculated by subtracting two times the amount of N_2_O formed from the initial amount of NO added to the reaction mixture, and standard deviation values for *f* ranged from 0.01–0.06 (Tables S1 and S2). ε_N‑bulk_ was determined by plotting δ^15^N against [*−f *ln *f*/(1 – *f*)] and determining the slope of this plot via linear regression (eq 4) (Figure S5B). In this type of plot, the reaction begins at 1 on the right side of the graph (all substrate present) and ends at 0 (no substrate remaining) on the left. To make the data presentation more intuitive, all graphs presented in the main text in this manuscript use a close approximation of [−*f* ln *f*/(1 – *f*)] for the *x*-axis, (1 – *f*), in which reaction progress proceeds from left to right. All calculations of ε_N‑bulk_, however, employ the full Rayleigh eq (eq 4) as described above.

Once ε is obtained, the KIE, the ratio of the reaction rate of the light over the heavy isotopically substituted compounds, can be calculated from the following equations

5 $$\varepsilon_{p/s}=\left(\alpha_{p/s}-1\right)\times 1000$$

6 $$\mathrm{KIE}=\frac{1}{\alpha_{p/s}}$$

7 $$\mathrm{KIE}=k_{L}/k_{H}$$

where α_p/s_ is defined as the fractionation factor (i.e., the instantaneous change in product *R* divided by substrate *R* (*R*_pi_/*R*_s_)) and *k*_L_ and *k*_H_ are the rate constants for the light and heavy isotopes, respectively.^54^

The fractionation factors for N^α^ and N^β^, α_N‑α_ and α_N‑β_, were determined using our recently described Expanded Rayleigh model (eqs 8–9),^48^ which more accurately accounts for position-specific isotopic fractionation than the standard Rayleigh model.

8 $$\alpha_{N-\alpha }=\frac{\rho }{\tau }\times \alpha_{N-\mathrm{bulk}}$$

9 $$\alpha_{N-\beta }=\frac{1-\rho }{1-\tau }\times \alpha_{N-\mathrm{bulk}}$$

Here α_N‑bulk_ is the bulk fractionation factor (determined using eqs 4 and 5), ρ is defined as ^15^N^α^/^15^N^bulk^, and τ is defined as ^14^N^α^/^14^N^bulk^. Like the standard Rayleigh model for product isotope ratios (eq 4), the Expanded Rayleigh model does not require that the initial substrate isotope ratio (δ^15^N^s0^) is known. The value of ρ was determined by nonlinear least-squares regression of eq 10

10 δN15bulk=12×[(ρ×N15bulk(N2Onmol−ρ×N15bulk)×Rstandard−1)×1000+δN15β]

following the method outlined by Baty et al.,^56^ with a starting value of ρ = 0.5. The value of τ was determined by averaging ^14^N^α^/^14^N^bulk^ for every observation. After α_N‑α_ and α_N‑β_ were calculated, these values were converted to position-specific enrichment factors (ε_N‑α_ and ε_N‑β_) and KIEs (KIE ^15^N^α^ and KIE ^15^N^β^) using eqs 5 and 6, respectively.

Site Preference (δ^15^N^SP^) was also calculated for the pooled replicates and is defined as

11 δN15SP=δN15α−δN15β

### Statistics and Figures

Data obtained prior to a (1 – *f)* value of 0.1 were excluded from analysis to avoid the slightly greater error associated with using Mariotti’s approximation of the Rayleigh eq (eq 4) for data collected near the start of the reaction.^55^ Data obtained after N_2_O production plateaued (i.e., after N_2_O concentration stopped increasing) were also excluded. After observations meeting either of these criteria were removed from each replicate, the data from replicates with the same experimental setup were pooled because the reaction mixtures were prepared from the same enzyme preparation and there was no reason to expect that the samples should behave differently from one another. To distinguish outliers in the data, the Grubbs’ test for one outlier^57,58^ was applied to the residuals from the linear regression of δ^15^N^bulk^ against [−*f *ln *f*/(1 – *f*)] for the data from pooled replicates. This test identifies the presence of an outlier based on the sample mean. (In this case, one outlier was identified among the data for N_2_O production in ^18^O enriched water, and one outlier was identified among the data for the corresponding experiment in unenriched water.)

Because the number of observations for each set of experiments was relatively small, bootstrapping was used to assess the goodness of fit for the standard Rayleigh (linear) model and Expanded Rayleigh model (nonlinear). Each pooled data set was sampled with replacement to generate 1000 data sets with the same number of observations as the original data set. KIE and ε values and root-mean-square error (RMSE) values were calculated for each resampled data set using the Rayleigh model (eq 4) and Expanded Rayleigh model (eqs 8 and 9) as described above, and the results were averaged (Table S3).

All statistics were performed in the statistical software package R (R Foundation, version 4.2.1),^59^ the Grubbs’ test was performed using the outliers package,^58^ and plots were produced using ggplot2.^60^

## Results

### Enzymatic N_2_O Production

We prepared three reaction mixtures containing NO and *P. denitrificans* cNOR in anaerobic serum bottles as described in the Material and Methods section, and we monitored the production of N_2_O by GC-ECD and/or IRMS (Figure 1). The rate of N_2_O production by cNOR was approximately 24 ± 2 nmol/min under our reaction conditions over the first 120 min of the reaction. Enzymatic activity began to plateau between approximately 3.0 and 3.4 μmol of N_2_O produced (58–66% substrate conversion). Isotope values (δ^15^N, δ^15^N^α^, δ^15^N^β^, and δ^18^O) and δ^15^N^SP^ (the difference in δ^15^N between the α and β nitrogen atoms in N_2_O) were measured by IRMS over an extent of reaction of approximately 20%–74% of substrate consumed (Table S1). This data was then utilized to calculate ε values and the corresponding KIEs (Table 1).

**Table 1:** Calculated^a^ Isotopic Enrichment Factors (ε), Kinetic Isotope Effects (KIEs), and Associated Statistics for N in the N_2_O Produced by *P. denitrificans* cNOR

measurement	ε (‰)^b^	KIE^b^	*R* ^2^ ^c^	linear RMSE^c^	nonlinear RMSE^d^	*p*-value (ε_N‑bulk_)^e^	*p*-value (ρ)^e^	*p*-value (τ)^e^
^15^N	–8.6 ± 0.9	1.0086 ± 0.0009	0.89	0.42	NA	1.33 × 10^–06^	NA	NA
^15^N^α^	–7.2 ± 1.0	1.0072 ± 0.0010	0.89	0.42	0.59	1.33 × 10^–06^	0.0022	0.0024
^15^N^β^	–9.9 ± 1.0	1.0100 ± 0.0010	0.89	0.42	0.59	1.33 × 10^–06^	0.0022	0.0024

a The three replicates were pooled into a single data set prior to analysis as described in the Materials and Methods section.

b ε or KIE value ± standard error.

c Value for linear regression of bulk δ^15^N (dependent variable) against [−*f *ln *f*/(1 *– f*)] (eq 4). *R* ^2^ = [1 – (SSR/SST)], where SSR is the residual sum of squares and SST is the total sum of squares. RMSE = root-mean-square error = √(SSR/*n*).

d RMSE value for nonlinear regression (eq 10) where bulk δ^15^N is the dependent variable.

e Null hypothesis: KIE = 1 (i.e., ε_N‑bulk_ = 0, or ρ = 0.5, or τ = 0.5). The parameters ρ and τ are only used to calculate position-specific isotope effects and thus are only listed for ^15^N^α^ and ^15^N^β^.

### Assessment of δ^18^O and δ^15^N of N_2_O Produced by *P. denitrificans* cNOR

Figure 2 illustrates the change in isotope ratio for both the oxygen and bulk nitrogen atoms of the product N_2_O as the reaction proceeds (Table S1). Linear regression of δ^18^O against reaction progress [−*f *ln f/(1 – *f*)] (i.e., application of the standard Rayleigh model, eq 4) yields a slope of −19.9 ± 1.9‰ (Figure S5A). The negative slope suggests a normal isotope effect (i.e., KIE > 1) in which the ^16^O isotope reacts more rapidly than the ^18^O isotope, leading to an enrichment of ^18^O in the residual substrate pool because ^16^O is incorporated into the product more quickly. As the reaction proceeds, both substrate and product become more enriched in the heavy isotope over time. It should be noted, however, that this slope is *not* the true enrichment factor (ε^18^O) as defined in eq 4 because it does not take into account the fractionation of the second substrate oxygen atom, which ends up in H_2_O.

**Figure 2:**
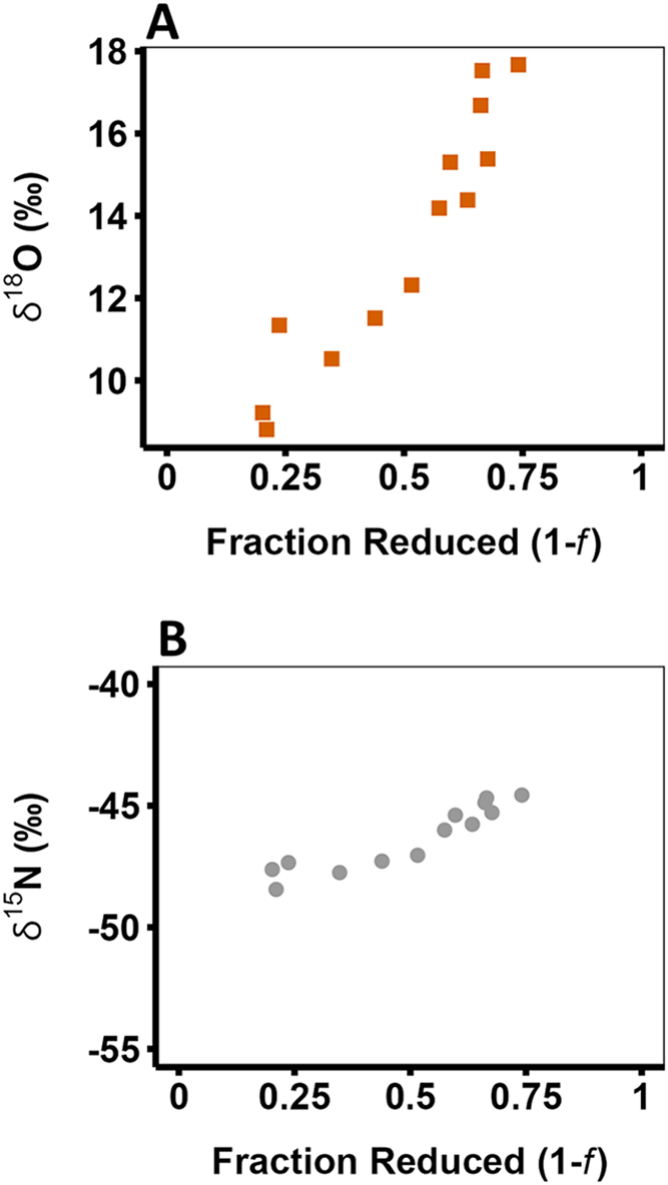
δ^18^O (A) and δ^15^N (B) of N_2_O produced by cNOR as a function of the fraction of substrate (NO) reduced (1 *– f*) with reactions progressing from left to right. The δ^18^O values are shown as orange squares, and the δ^15^N values are shown as gray circles. The data from three replicate experiments were pooled; each data point represents one IRMS measurement. A linear plot of the data fit to the Rayleigh eq (eq 4) is provided in Supporting Figure S5.

An experiment was also performed with water enriched in ^18^O to examine the possibility of isotopic exchange of oxygen between water and NO (Figures S1–S4 and Table S2). If exchange occurred, there would be an offset in the δ^18^O value of the N_2_O produced. As evident in Figure S2A, there does not appear to be a significant offset in the δ^18^O values between the ^18^O spiked and nonspiked replicates, suggesting that if any isotopic exchange of oxygen occurred under our reaction conditions, it was below our detection limit and can therefore be ignored. Although the *f* range of this control experiment was too small to accurately measure the slope, the general increase in δ^18^O as the reaction progressed is further indication of a normal isotope effect for ^18^O during N_2_O production.

The δ^15^N of N_2_O has an ε value of −8.6 ± 0.9‰ with an increase in δ^15^N slightly less than 4‰ over the measured reaction (Figures 2B and S5B and Table 1, *R* ^2^ = 0.89). Importantly, the linearity of the increase in δ^15^N values means that the standard Rayleigh eq (eq 4) can be used to accurately determine ε. The bulk KIE ^15^N value was 1.0086 ± 0.0009, indicating that the reaction rate for ^14^NO is faster than the rate for ^15^NO. The initial δ^15^N value of the substrate (NO), δ^15^N^s0^, (eq 4) was calculated to be −40.5‰.

### δ^15^N^α^, δ^15^N^β^, and δ^15^N^SP^ Values for N_2_O Produced by *P. denitrificans* cNOR

The δ^15^N^α^ values remained essentially constant over the course of the reaction (Figure 3A and Table S1), ranging from −42‰ to −46‰. All of the δ^15^N^α^ values are less than the calculated δ^15^N^s0^ value for NO (−40.5‰), indicating that the α position in N_2_O is enriched in ^14^N relative to the initial substrate, which suggests that N^α^ is subject to a normal isotope effect. The lack of an apparent trend in δ^15^N^α^ values appears to be an artifact due to noise in the data. Despite this noise in δ^15^N^α^ values, when the nonlinear portion of the Expanded Rayleigh model (eq 10) is applied to the isotopic data for N (δ^15^N^α^, δ^15^N^β^, and δ^15^N), the RMSE value (0.59) is similar to the RMSE value obtained when the standard Rayleigh model (eq 4) is applied to δ^15^N values (0.42), indicating that both models have a similar goodness of fit. Thus, the Expanded Rayleigh model can be used to determine position-specific enrichment factors with a level of accuracy similar to that of the bulk enrichment factor (ε_N‑bulk_), which is calculated using the standard Rayleigh model.[Fn fn4] Calculation of the KIE for ^15^N^α^ using the Expanded Rayleigh model yields a normal KIE value of 1.0072 ± 0.0010 and an ε value of −7.2 ± 1.0‰ (Table 1), indicating that ^14^N is incorporated into the α position more quickly than ^15^N.

**Figure 3:**
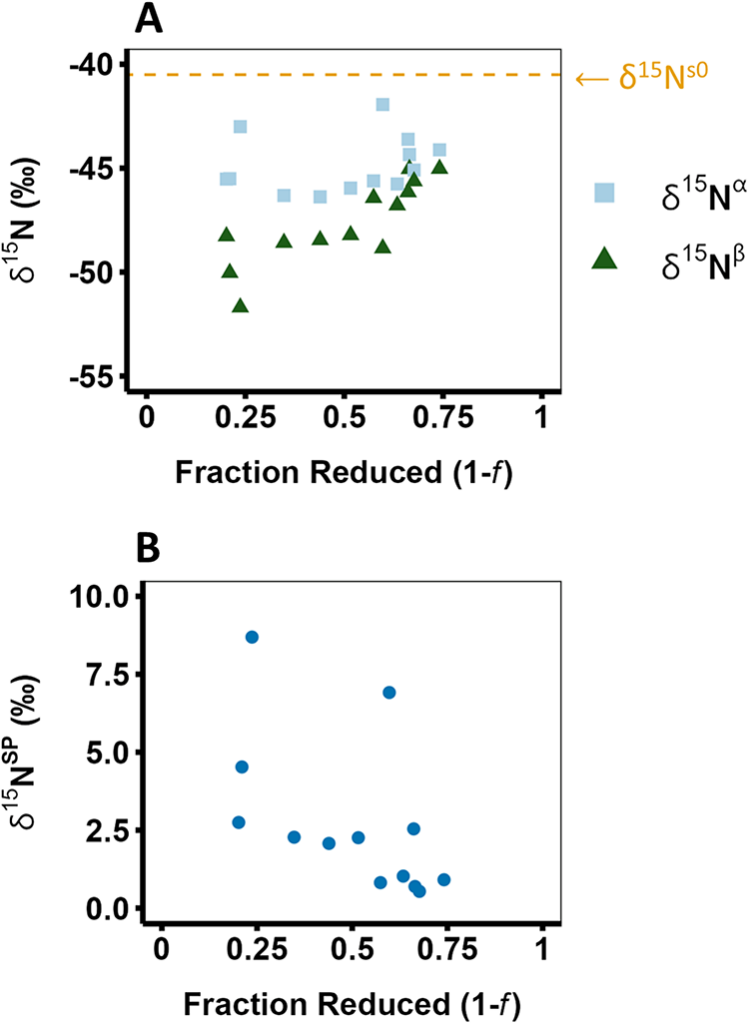
δ^15^N^α^ and δ^15^N^β^ (A) and δ^15^N^SP^ (B) of N_2_O produced by cNOR as a function of the fraction of substrate (NO) reduced (1 – *f*) with reactions progressing from left to right. δ^15^N^α^ values are shown as light blues squares, δ^15^N^β^ values are shown as dark green triangles, and δ^15^N^SP^ values are shown as blue circles. The calculated initial substrate δ^15^N value (δ^15^N^s0^, the *y*-intercept of eq 4 when δ^15^N^p^ = δ^15^N^bulk^) is shown as an orange dashed line in panel (A). The data from three replicate experiments were pooled; each data point represents one IRMS measurement. The δ^15^N^SP^ values shown in (B) were plotted against [−*f *ln* f*/(1 – *f*)] in Supporting Figure S6, and it was determined that there is no significant trend in δ^15^N^SP^.

The δ^15^N^β^ values are calculated using δ^15^N values and δ^15^N^α^ values (i.e., by assuming that bulk δ^15^N is equal to the average of δ^15^N^α^ and δ^15^N^β^),^51^ and the trend in δ^15^N^β^ values is therefore a combination of the trends in δ^15^N and δ^15^N^α^. The δ^15^N^β^ values increased over the course of the reaction, exhibiting a range of 7‰ over the measured reaction (Table S1). Similar to the δ^15^N^α^ data, the δ^15^N^β^ values were all less than the calculated δ^15^N^s0^ value, qualitatively indicating that ^14^N is also enriched at the β position of N_2_O (normal isotope effect). In agreement with this qualitative assessment, a normal KIE of 1.0100 ± 0.0010 and ε value of −9.9 ± 1.0‰ were calculated for ^15^N^β^ (Figure 3A and Table 1). Thus, ^14^N is also incorporated into the β position more rapidly than ^15^N is incorporated. Additionally, the fact that KIE ^15^N^β^ is slightly larger than KIE ^15^N^α^ indicates that the difference in the reaction rates for the light and heavy isotopes is more pronounced at the β position. The control experiment with H_2_^18^O indicated the same isotopic trends for δ^15^N^α^ and δ^15^N^β^ as discussed above (Figure S3).

Because the number of observations for cNOR was relatively small (13 observations from 3 replicates), we performed a bootstrapping analysis of the data to validate the calculated KIE values. Averaging the KIE values from 1000 bootstrapped data sets yielded a KIE ^15^N value of 1.0087 ± 0.0010, a KIE ^15^N^α^ value of 1.0073 ± 0.0012, and a KIE ^15^N^β^ value of 1.0101 ± 0.0009 (Table S3). Thus, resampling the original data with replacement produced results that were very similar to the original calculated data set.

From *in vivo* studies of N_2_O production, δ^15^N^SP^ has been found to be nearly constant over time,^61,62^ making it a valuable tool for distinguishing N_2_O derived from nitrification versus denitrification.^63,64^ In the current study, we have a closed system with a finite pool of substrate. If the expressed KIEs for the α and β positions remain constant over the course of the reaction, we would expect δ^15^N^SP^ to remain fairly constant as well.^48^ Indeed, no significant trend in δ^15^N^SP^ values is observed over the course of the measured reaction (Figures 3B and S6). The δ^15^N^SP^ values range from as high as 8.7‰ near the start of the reaction to as low as 0.5‰ near the end of the reaction, with an average value of 2.8 ± 2.5‰. Although these values appear to decrease as the reaction proceeds, the correlation between δ^15^N^SP^ values pooled from all three replicates and [-*f*ln*f*/(1-*f*)] is poor (R^2^ = 0.29, Figure S6). The fact that these values are all fairly close to 0‰ indicates that the enrichment factors for N^α^ and N^β^ are very similar, in agreement with our calculated position-specific KIEs (Table 1).

## Discussion

### Considerations of Observed KIEs

In this manuscript, we measured the observed KIEs for N^α^, N^β^, and ^18^O to gain mechanistic insights into cNOR from *P. denitrificans*. It is important to note that all KIEs presented in the manuscript are observed KIEs (KIE_obs_) as opposed to intrinsic KIEs (KIE_int_), which are defined as the inherent isotope effect for a specific step in a reaction.^65,66^ Conversion of a substrate to a product by an enzyme inherently involves multiple steps, each of which could have a unique isotope effect. Typically, chemical steps (bond breaking or formation) have the largest KIE_int_ values, although substrate binding (pre-equilibrium) can also exhibit significant equilibrium isotope effects (EIEs) under certain circumstances. Thus, both intrinsic EIE values (EIE_int_) and KIE_int_ values can contribute to the KIE_obs_ value measured for an enzyme-catalyzed reaction.

The extent to which each EIE_int_ or KIE_int_ value affects KIE_obs_ depends on the magnitude of each intrinsic isotope effect and the relative energy barrier of each TS. Any reaction step up to and including the first irreversible step can have an impact on KIE_obs_, and steps with higher energy barriers will be more influential.^42,65,66^ Thus, KIE_obs_ depends on a complex mixture of the various EIE_int_ and KIE_int_ values in a reaction sequence. Because KIE_int_ values for chemical steps are typically larger than the KIE_int_ values for processes that do not involve changes in substrate bond order (e.g., diffusion of gaseous substrate into solution or protein conformational changes), KIE_obs_ typically trends toward a value of one relative to the KIE_int_ of a specific chemical step. In other words, KIE_obs_ can typically be considered a lower limit of KIE_int_ for a chemical step with a normal isotope effect (KIE > 1) and an upper limit for a chemical step with an inverse isotope effect (KIE < 1).^44,67^

It is also important to note that other reactions can influence the isotope ratio of the enzymatically produced N_2_O. One such reaction is the exchange of oxygen between water and NO. Results from monoculture experiments suggest that exchange of oxygen occurs during either the reduction of nitrite (NO_2_^–^) to NO or the conversion of NO to N_2_O.^68^ The lack of oxygen exchange observed in our experiments indicates that, at least for *P. denitrificans*, the oxygen exchange observed in microbial studies must occur before conversion of NO to N_2_O (Figure S2A).

Other reactions to consider that might impact our isotope data are any processes that consume or produce either NO or N_2_O. For example, NO can react with O_2_ to form NO_2_. Due to the highly reactive nature of NO and the fact that ^15^N^15^N has the same mass as ^14^N^16^O, we did not quantify NO concentration or δ values in this study. Our reactions, however, were performed under a rigorously controlled anaerobic environment, minimizing the possibility that reactions involving O_2_ contributed in any significant way to our results. If the reduction of NO to N_2_O were reversible, this would also alter the apparent isotope effect of N_2_O production. At pH 7.0, however, the standard reduction potential of NO → N_2_O is +1.18 V, indicating that this reaction is highly exergonic and unlikely to be easily reversed,^69^ and there is no indication in the current literature that this reaction is reversible. In addition, we are not aware of any examples of cNOR further reducing N_2_O to N_2_. Another way N_2_O could be produced is via codenitrification, a process in which inorganic nitrogen (NO_3_^–^ or NO_2_^–^) reacts with an organic source, such as an amino acid.^70^ Due to the reducing conditions of our reaction and the sources of nitrogen available (NO, TMPD, and the buffer), it is highly unlikely that codenitrification is taking place. Finally, control experiments in the absence of cNOR did not produce N_2_O levels above background, indicating that the contribution from nonenzymatically produced N_2_O is minimal (Figure 1).

### Insights from Site Preference

There are multiple biotic and abiotic processes that can produce N_2_O, and, owing to mechanistic differences, these processes often yield N_2_O with distinct δ^15^N^SP^ values. In particular, studies of bacterial cultures examining N_2_O production during denitrification or nitrifier-denitrification demonstrate that the N_2_O produced under such conditions has relatively low δ^15^N^SP^ values (i.e., δ^15^N^SP^ ≈ 0‰).^62,64,71^ Similarly, our experiments show that purified *P. denitrificans* cNOR produces N_2_O with low δ^15^N^SP^ values throughout the measured reaction (average δ^15^N^SP^ = 2.8 ± 2.5‰; range = 0.5–8.7‰), in agreement with previous single time point measurements for N_2_O produced by purified *P. denitrificans* cNOR (average δ^15^N^SP^ = −5.9 ± 2.1‰).^72^ These *in vitro* studies support the hypothesis that N_2_O biosynthesis catalyzed by cNOR (or a phylogenetically related respiratory NOR) is the dominant source of N_2_O released during bacterial denitrification. It should be noted, however, that flavohemoglobin, which produces N_2_O with δ^15^N^SP^ values of ∼10‰, may also be a minor source of N_2_O produced during bacterial denitrification.^20^

In addition to confirming that cNOR-catalyzed N_2_O biosynthesis yields N_2_O with low δ^15^N^SP^ values, our data also show that δ^15^N^SP^ appears to remain fairly constant as the fraction of substrate consumed (1– *f*) increases from 20% to 74%. Thus, in contrast to N_2_O biosynthesis by purified fungal P450nor,^47^ the δ^15^N^SP^ values for N_2_O produced by purified cNOR are largely independent of substrate concentration. The lack of a trend for δ^15^N^SP^ in the present study indicates that the observed isotope effects for N^α^ and N^β^ remain constant as *f* varies, allowing us to use the Expanded Rayleigh model to calculate KIE ^15^N^α^ and KIE ^15^N^β^.^48^

### Physical Basis for Isotope Effects

As discussed above, the KIE_obs_ values measured in this study likely arise from a combination of EIE_int_ values (due to substrate binding pre-equilibria) and KIE_int_ values for all reaction steps starting with substrate binding through the first kinetically irreversible step. For example, binding of each NO molecule is expected to affect KIE_obs_ of either N^α^ or N^β^. For NO coordination to Fe_B_, binding and dissociation both appear to be fairly rapid,^37,38^ so this binding step is likely a pre-equilibrium process with an EIE_int_. On the other hand, *k*_on_ is typically several orders of magnitude greater than *k*_off_ when NO binds to reduced heme,^15^ suggesting that this binding step may be governed by a KIE_int_. Subsequent steps such as N–N bond formation also have the potential to influence KIE_obs_ at both the α and β positions. Thus, understanding the physical basis of KIE_obs_, which can lead to mechanistic insights, depends on understanding the physical basis of both EIEs and KIEs.

The physical basis for an EIE can be described as the product of three energetic terms expressed as reduced partition functions: mass and moments of inertia (MMI), the energy of excited vibrational states (EXC), and zero-point energy (ZPE).^73–75^

12 EIE=MMI×EXC×ZPE

Each of the terms in eq 12 is defined as a ratio (final state/initial state) of isotopologous ratios using vibrational frequencies for the heavy and light isotopes.^74,76^ Similarly, KIE can be defined as a special type of EIE where the final state is the TS (and MMI, EXC, and ZPE have one fewer vibrational frequency for the TS):^43,74–76^

13 KIE=νLRCνHRC×MMI×EXC×ZPE=TSDF×MMI×EXC×ZPE

In eq 13, ^L^ν_RC_ and ^H^ν_RC_ refer to the imaginary frequency along the reaction coordinate for the bond(s) being broken/formed for the light and heavy isotopes, sometimes referred to as the transition state decomposition frequency (TSDF).^74,76^ Thus, using the formalisms in eqs 12 and 13, theoretical EIEs and KIEs can be completely described as a function of the differences in vibrational frequencies between bonds for the light and heavy isotopes in the initial state and the final state.

It is commonly assumed that isotope effects (EIEs or KIEs) are due primarily to differences in zero-point energy levels between bonds comprised of the lighter and heavier isotopes in the initial and final states.^74^ When ZPE is the dominant contributor to isotope effects, the heavy isotope is enriched in the more tightly bound state. In heavy-atom (e.g., N or O) isotope effects, however, the zero-point energy difference between the light and heavy atoms is relatively minor, and EXC, MMI, and TSDF may contribute substantially to the observed EIE and/or KIE.^74^ Indeed, Roth and colleagues have shown that, at or near room temperature, EXC*MMI is the dominant contributor for metal-mediated O_2_ binding to reduced metals,^77–79^ and these terms are also expected to play a significant role in determining KIEs for O_2_ activation.^79^ Additionally, although TSDF is approximately equal to one in some cases,^80^ in other cases TSDF can be significantly greater than one.^78,81^ Thus, while it was previously assumed^47,82,83^ that ZPE is the dominant factor determining isotope effects for interactions of small, gaseous molecules like O_2_ or NO with metals, this assumption has been shown to be inaccurate for O_2_ binding^79^ and is likely also an inadequate description of NO binding to metals. Analogous to O_2_ binding to reduced metals, however, NO appears to become formally reduced during all potential NO binding steps proposed for cNOR (see below). Therefore, it may be reasonable to assume, as a first approximation, that the EIEs or KIEs for NO binding to Fe­(II) in cNOR are also normal.

Calculating theoretical EIE_int_ and KIE_int_ values for each of the proposed catalytic mechanisms for cNOR requires determining all isotopically sensitive vibrational frequencies for the initial and final states for all relevant steps in each mechanism, including theoretical transition states.^78,79^ While a few Fe-NO or N–O vibrational frequencies have been measured experimentally for cNOR or proteins/models with similar bimetallic centers, many of these frequencies would have to be determined computationally. Such calculations are beyond the scope of this study. However, the isotopically sensitive steps that are expected to contribute to KIE_obs_ for each mechanism are outlined below, along with a qualitative assessment of how the expected binding EIEs or KIEs for each mechanism compare with our experimental results. For simplicity, we will limit our discussion to primary isotope effects (i.e., effects for making or breaking a bond to an isotopic atom), which are larger than secondary isotope effects.

### Mechanisms and Mechanistic Insights

Currently there are three distinct mechanisms proposed for the production of N_2_O by cNOR (Scheme 3), with varying levels of evidence. All three mechanisms start with NO binding to the fully reduced enzyme but differ in where NO binds. The *cis*-Fe_B_ mechanism proposes that the first NO binds the nonheme Fe_B_ site. Originally, it was proposed that the second NO molecule also binds Fe_B_ to form a dinitrosyl species [Fe_B_-(NO)_2_],^85^ but this version of the mechanism has been largely ruled out because N–N bond formation would be spin forbidden and thus prohibitively high in free energy.^86^ Alternatively, the second NO could directly attack the NO bound to Fe_B_, forming a hyponitrite intermediate with one N atom coordinated to Fe_B_ (Scheme 3, top).^34,35^ In this mechanism, heme *b*_3_ would participate in electron transfer without binding NO.^34,84^ In contrast, the *cis*-heme *b*_3_ mechanism argues that the first NO binds to heme *b*_3_ and the second NO then attacks the first NO to form the N–N bond.^35,41,87–89^ In this scenario, the nonheme Fe_B_ would act as a Lewis acid, and coordination of the O atom of the heme *b*_3_-nitrosyl complex to Fe_B_ is thought to activate the bound NO for direct attack by the second NO.^15,35^ DFT calculations suggest that the resulting *cis*-hyponitrite intermediate is a five-membered ring with one N atom coordinated to Fe_*b*3_ (i.e., the iron center of heme *b*_3_) and both O atoms coordinated to Fe_B_; this intermediate then rotates to form a second ring intermediate with one O atom bridging Fe_B_ and Fe_*b*3_.^35,41,88^ Finally, the *trans* mechanism proposes that one NO molecule binds to each Fe in the active site, followed by formation of the N–N bond by radical–radical coupling or electrophilic attack.^15,29,36–38,90^

**Scheme 3:**
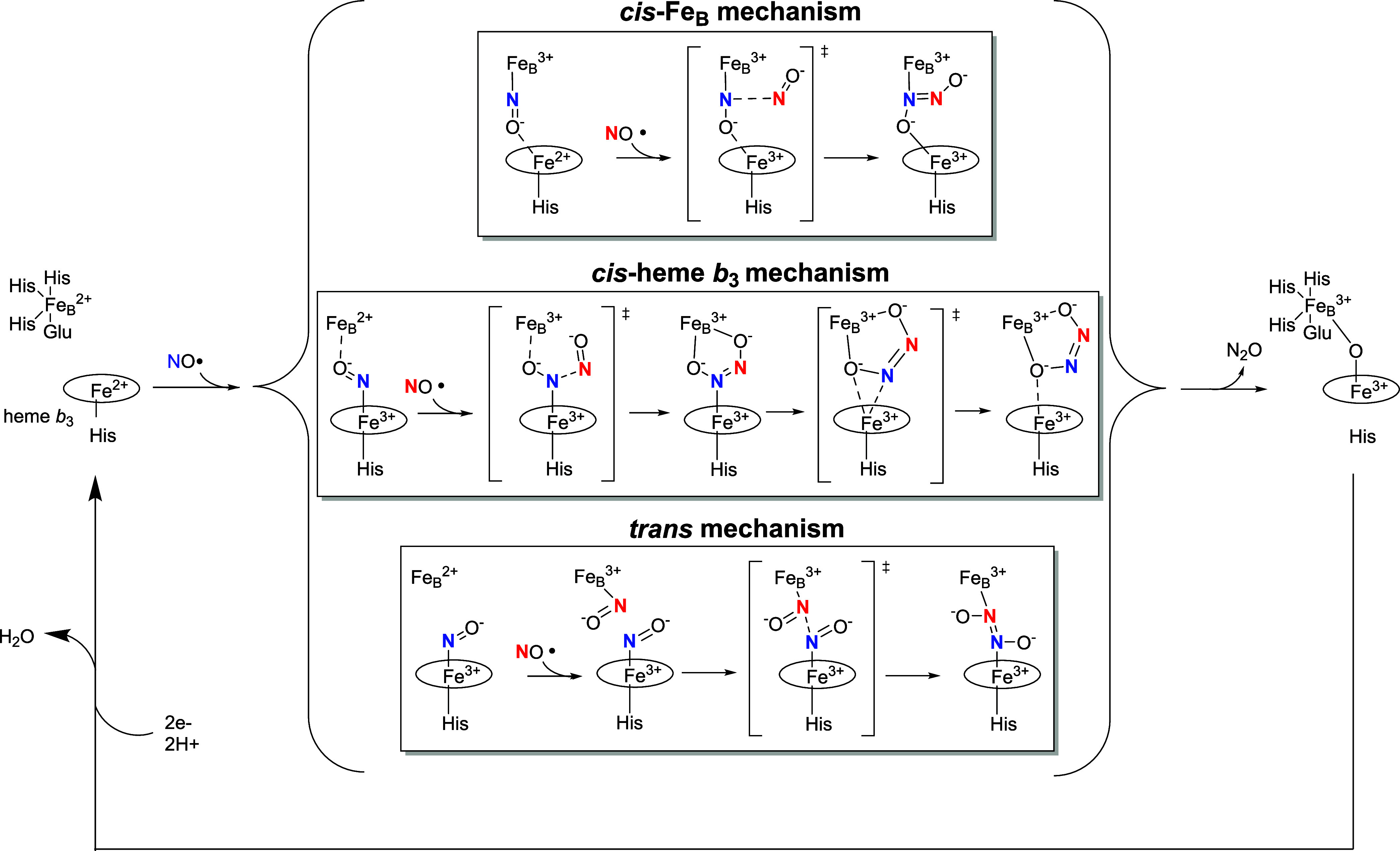
Three Distinct Proposed Mechanisms of cNOR, Adapted from Moënne-Loccoz^84^ and Blomberg^a^ ^35^

a All Fe-nitrosyl ({FeNO}^7^) complexes are shown as Fe­(III)-NO^–^ as this formal oxidation state is likely the best description of each of these complexes (see Discussion).

All three mechanisms predict a hyponitrite species as an intermediate, albeit with different structures/coordination (Scheme 3), and N_2_O release requires breaking an N–O bond in this intermediate. Although there is no general consensus on the exact mechanism by which hyponitrite loses an oxygen to form N_2_O, one compelling possibility for the *cis*-heme *b*_3_ mechanism is that one of the oxygen atoms remains in the active site bound to both metal ions as the bridging oxo found in the resting state of the enzyme [Fe_*b*3_(III)–O-Fe_B_(III)].^32,33^

There is still much debate about which mechanism is most accurate for cNOR. Spectroscopic evidence indicates that NO can bind to ferrous heme *b*_3_ as well as ferrous Fe_B_ under single-turnover conditions.^31,36–39,89^ Evidence of NO binding to reduced Fe_*b*3_ seems to argue against the *cis*-Fe_B_ mechanism but is potentially consistent with either the *cis*-heme *b*_3_ or *trans* mechanisms. Data showing that both Fe centers in cNOR can bind NO is generally interpreted as support for the *trans* mechanism. However, not all NO binding is necessarily catalytically relevant, and a hyponitrite intermediate has not been observed under turnover conditions, precluding a definitive mechanistic interpretation.^31,36–39^ More recently, time-resolved spectroscopic studies have shown that the first NO binds Fe_B_(II) and then migrates to Fe_*b*3_(II) before the second NO molecule enters the active site and N_2_O is formed. As it is not clear if/where the second NO molecule binds prior to N–N bond formation, this data can also be interpreted in favor of either the *cis*-heme *b*_3_ or *trans* mechanisms. Additional evidence for the *trans* mechanism comes from a myoglobin variant that was engineered to mimic the active site of cNOR by adding a nonheme Fe_B_ site near the heme (Fe_B_Mb).^91^ Lin et al. provided evidence that, at least in this model system, N_2_O production proceeded through a *trans* mechanism.^90,92^ On the other hand, computational investigations strongly favor the *cis*-heme *b*_3_ mechanism,^35,88^ and recent flow-flash experiments with cNOR are consistent with the computational predictions for this mechanism.^89^ Thus, despite recent advances, the precise details of the catalytic mechanism of cNOR remain unresolved.

In this study, we examined the isotopic enrichment of bulk ^15^N, ^15^N^α^, ^15^N^β^, and ^18^O in N_2_O produced by purified cNOR at multiple time points (i.e., at multiple NO concentrations/values of *f*). The δ^18^O values we measured for N_2_O increased (i.e., became more enriched in ^18^O) as the reaction proceeded. Unfortunately, this trend is difficult to interpret mechanistically without data on the initial substrate δ^18^O value (which is exceedingly challenging to measure due to the inherent reactivity of NO) or the δ^18^O fractionation for the NO oxygen atom that is incorporated into water (which is also unknown). The enrichment of ^18^O in N_2_O over time, however, suggests that for at least one of the substrate O atoms, ^16^O reacts faster than ^18^O. Thus, NO reduction to N_2_O by cNOR may contribute to the enrichment of ^14^N^14^N^18^O during bacterial denitrification (conversion of nitrate to N_2_O), a trend which has been observed in some axenic cultures.^62^ It should be noted, however, that *depletion* of ^18^O in N_2_O has been seen in other studies of bacterial denitrifiers,^64,71^ indicating that the isotope effect from cNOR can be overshadowed by isotopic exchange that occurs during the other reactions or diffusion processes involved in denitrification.

Our isotopic data for bulk N and the individual α and β positions in N_2_O clearly show that there is a normal isotope effect for N^bulk^, N^α^, and N^β^, with the isotope effect at N^β^ being slightly greater than the isotope effect at N^α^. Broadly speaking, because NO is expected to bind via coordination of the N atom to Fe_B_ or Fe_*b*3_, the observed isotope effects for the N atoms in N_2_O are likely a combination of binding isotope effects (pre-equilibrium EIEs or KIEs) and kinetic isotope effects due to the isotope-sensitive step(s) of catalysis that occur up through the first irreversible step.^35,88^ For cNOR, it is reasonable to assume that the first irreversible step in each of the three proposed mechanisms is N–N bond formation, although more detailed calculations for the *cis*-heme *b*_3_ mechanism suggest that the first irreversible step could also be rotation of the *cis*-hyponitrite intermediate. The expected isotope effects for the isotopically sensitive steps from each mechanism are outlined below.

#### ^15^N Isotope Effects in the *cis*-Fe_B_ Mechanism

To form N_2_O via the *cis*-Fe_B_ mechanism, one NO must bind to Fe_B_(II), followed by the attack of a second NO on the bound NO to form an N–N bond. Binding of NO to a nonheme, high-spin Fe­(II) center typically results in a Fe-nitrosyl complex with Fe­(III)-NO^–^ character.^15^ Indeed, time-resolved EPR and IR spectroscopic data for the transient {Fe_B_NO}^7^ complex formed when NO binds to Fe_B_(II) are consistent with increased π* electron density for the bound NO, indicating that Fe­(III)-NO^–^ is the dominant resonance structure.^38^ Because NO is formally reduced upon binding Fe_B_(II), similar to the formal reduction of O_2_ upon coordination to a reduced metal,^79^ NO binding to Fe_B_(II) may have a normal isotope effect. Thus, the normal KIE that we observe for one of the N atoms in N_2_O could be due to NO entering the empty, reduced binuclear active site and binding Fe_B_, which would be consistent with the *cis*-Fe_B_ mechanism. In this scenario, KIE_obs_ for the second, attacking NO molecule would presumably be due to the kinetic isotope effect associated with N–N bond formation, although without a TS structure, it is difficult to predict the type of KIE associated with this step. However, DFT calculations for the proposed intermediate in this mechanism suggest that formation of this intermediate is energetically unfeasible.^35^ This prediction, together with the general lack of evidence for this mechanism and multiple studies showing that NO rapidly binds reduced heme *b*_3_,^31,36,37,39^ lead us to regard the *cis*-Fe_B_ mechanism as unlikely.

#### ^15^N Isotope Effects in the *cis*-Heme *b*_3_ Mechanism

To form N_2_O via the *cis*-heme *b*_3_ mechanism, the first NO molecule must bind ferrous heme *b*_3_, forming either a 5-coordinate (5C)^36^ or 6-coordinate (6C)^39^ {FeNO}^7^ complex, followed by attack of the second NO to form a *cis*-hyponitrite intermediate that is proposed to be a five-membered ring with both O atoms coordinated to Fe_B_ (Scheme 3).^35,41,88^ Experiments with a 5C {FeNO}^7^ synthetic porphyrin complex^93^ and a 6C {FeNO}^7^ protein model of cNOR (Fe_B_Mb)^94^ both suggest that positioning a Lewis acid (e.g., nonheme Fe) near the heme gives the ferrous heme nitrosyl complex more Fe­(III)–NO^–^ character. Thus, regardless of whether NO binding to heme *b*_3_ yields a 5C or 6C species, NO binding to heme *b*_3_ may produce a normal isotope effect. Furthermore, detailed hybrid DFT calculations for the *cis*-heme *b*_3_ mechanism suggest that the NO molecule that binds heme *b*_3_ is the source of the β nitrogen^35,88,95^ (assuming the hyponitrite intermediate is not released prior to N–O bond cleavage). Therefore, our data, which indicate a normal KIE for N^β^, are consistent with this part of the *cis*-heme *b*_3_ mechanism.

After formation of a heme *b*_3_-nitrosyl complex, the next step in the *cis*-heme *b*_3_ mechanism is formation of the N–N bond via electrophilic attack by a second NO molecule.^35^ Blomberg predicted the TS for N–N bond formation via the *cis*-heme *b*_3_ mechanism using hybrid DFT calculations.^35,88^ The predicted TS appears to be reasonably early, as the second NO is still fairly distant (∼2 Å) from the bound N of the heme nitrosyl complex and the other bond lengths for the bound and free NO are only slightly altered in the TS.^35,88^ Based on this TS where the bonding modes for each N atom are essentially the same as in the ground state, it seems unlikely that the attacking N atom (likely N^α^) would have a KIE significantly different from unity. Additionally, although the next reaction step, *cis*-hyponitrite rotation, is also potentially isotopically sensitive, only N^β^ is expected to experience a primary isotope effect during this step (due to the loss of an Fe_*b*3_-N bond in the TS).^88^ Thus, while N^β^ may be subject to a normal KIE during NO binding and during *cis*-hyponitrite rotation, N^α^ is not expected to experience a significant isotope effect in the *cis*-heme *b*_3_ mechanism. In contrast, our data indicate that both N atoms are subject to normal KIEs.

One can envision two possible explanations for this discrepancy. In one scenario, the second NO would transiently bind Fe_B_(II) before dissociating and attacking the NO bound to heme *b*_3_ (Scheme S1). The pre-equilibrium with Fe_B_ could effectively increase the concentration of ^14^NO in the active site relative to ^15^NO, causing the second N atom to have a normal KIE_obs_ (see below). Alternatively, the TS for N–N bond formation may not be as early as the published DFT calculations suggest, or the assumption that TSDF is very close to unity may be flawed (see eq 13). Generation of the *cis*-hyponitrite intermediate involves the rotation of the bound NO, formation of the N–N bond, and oxidation of both iron centers,^35,41,88^ making this chemical step a complicated process to model. Overall, the *cis*-heme *b*_3_ mechanism is consistent with our data for N^β^, but it is less clear how the normal KIE that we observe for N^α^ would be produced. Due to the complexity of the cNOR-catalyzed reaction, however, we are hesitant to rule out this mechanism without further computational analysis.

#### ^15^N Isotope Effects in the *trans*-Mechanism

Finally, producing N_2_O via the *trans* mechanism requires binding one NO molecule to each reduced Fe in the active site, followed by N–N bond formation. As discussed above for the *cis*-Fe_B_ and *cis*-heme *b*_3_ mechanisms, when starting from the empty, reduced binuclear center, NO binding to either Fe_B_ or Fe_*b*3_ may have a normal isotope effect, in analogy to O_2_ binding reduced metals. Additionally, if the first NO molecule initially binds Fe_B_ before dissociating and binding Fe_*b*3_, as time-resolved spectroscopy suggests,^37,38^ this could increase the normal KIE ^15^N for the first N atom, as enrichment for ^14^N will occur during each binding event.

Assuming that the first NO molecule ultimately ends up bound to heme *b*_3_,^37,38^ the *trans* mechanism predicts that the second NO molecule will bind Fe_B_(II). In the presence of a heme *b*_3_-nitrosyl, the resulting Fe_B_-NO complex is expected to be a typical nonheme {FeNO}^7^ complex with Fe­(III)-NO^–^ character but without the unusually high π* electron density seen in the absence of the heme *b*_3_-nitrosyl.^38^ Studies of similar diiron dinitrosyl complexes^92,96,97^ indicate that NO binding to Fe_B_ in the presence of heme *b*_3_-NO will result in formal reduction of NO, suggesting that the binding isotope effect is normal in analogy to metal-O_2_ coordination EIEs. Thus, the normal KIEs observed for N^α^ and N^β^ may be consistent with one NO molecule binding each active site Fe center, as predicted by the *trans* mechanism.

Formation of the N–N bond via the *trans* mechanism is generally proposed to occur via radical–radical coupling or electrophilic attack.^15,36^ While DFT calculations suggest that the *trans*-hyponitrite intermediate is prohibitively high in energy,^35,88^ the TS structure has not been predicted, making it difficult to assess what type of isotope effect would be associated with *trans*-hyponitrite formation. Thus, additional theoretical studies are needed to determine if the normal KIEs we observe for N^α^ and N^β^ are consistent with the combination of NO binding steps and N–N bond formation predicted for the *trans* mechanism.

## Conclusions

In summary, our isotopic data demonstrate that both N^α^ and N^β^ are subject to a normal isotope effect during N_2_O biosynthesis. The bulk N isotope effect is also normal, indicating that cNOR-catalyzed N_2_O biosynthesis contributes to the depletion in ^15^N observed for N_2_O produced by bacterial denitrifiers.^62,64,71^ Also consistent with studies of axenic cultures of bacterial denitrifiers,^20,62,64,71^ we found that the δ^15^N^SP^ values of N_2_O produced by cNOR were close to 0‰, with no significant trend as substrate was consumed. Additionally, the enrichment in N_2_^18^O observed as the reaction proceeds suggests that at least one of the substrate O atoms is subject to a normal isotope effect. *This is the first time N*_*2*_ *O biosynthesis by purified cNOR has been examined at multiple time points, and, more importantly, the first study where the position-specific isotope effects for N* ^α^ *and N* ^β^ *have been measured for N*_*2*_ *O produced by a respiratory bacterial NOR*.

Interpretation of the KIEs we measured for N^α^ and N^β^ is hindered by a lack of data on how the changes in N–O and Fe-NO bonding and vibrational frequencies will interact to produce KIE_obs_. Based on analogy with the EIEs and KIEs calculated for O_2_ binding to reduced metal complexes,^77–81^ coordination of NO to either Fe_*b*3_(II) or Fe_B_(II) may produce a normal isotope effect. Thus, our data appear to be consistent with the first part of the *trans* mechanism, which includes two NO binding steps. Additionally, our data are also potentially consistent with the *cis*-Fe_B_ and *cis*-heme *b*_3_ mechanisms for at least the N atom that binds an Fe­(II) center.

Overall, our isotopic data provide important constraints for evaluating proposed catalytic mechanisms for cNOR. As discussed above, each mechanism predicts the formation of one or more {Fe-NO}^7^ complexes with unique electronic structures, which could potentially give rise to EIE_int_ and KIE_int_ values with distinct magnitudes. Fully unraveling the mechanistic implications of the KIEs measured for cNOR will require measurement or computational prediction of EIEs or KIEs for each proposed NO binding step, as well as careful theoretical analysis of the TS and corresponding KIE_int_ for each of the appropriate reaction steps. Additional measurements of vibrational frequencies for Fe-NO complexes in cNOR or relevant models will also be critical for benchmarking theoretical calculations.

## Supplementary Material



## Data Availability

A Zenodo repository with the data and code used in this study is available at 10.5281/zenodo.17069751.

## Footnotes

1 The global warming potential of N_2_O with and without inclusion of the climate-carbon feedback in response to emitted N_2_O is estimated to be 298 times and 265 times greater than that of CO_2_, respectively.^3^ Climate-carbon feedback refers to the effect of changes in climate (e.g., changes due to N_2_O emissions) on the carbon cycle, which affects atmospheric CO_2_ concentrations.^4^

2 In the US, 75% of anthropogenic N_2_O emissions are due to agricultural soil management, and an additional 4% of these emissions are due to manure management.^7^

3 Formation of a ferric heme *b*_3_-NO complex in NOR, followed by addition of reductant, led to the formation of a hyponitrite species^40^ that appears to be *cis*-hyponitrite.^35,41^ It remains unresolved, however, if this is the same hyponitrite species formed during catalytic turnover.

4 Because the (linear) standard Rayleigh model (eq 4) and the nonlinear portion of the Expanded Rayleigh model (eq 10) both use δ^15^N as the dependent variables, the RMSE values for both models can be compared directly.

## Notes

Figure S1 displays enzymatic N_2_O production with and without H_2_^18^O enriched water; Figures S2–S4 display δ values (δ^18^O, δ^15^N, δ^15^N^α^, and δ^15^N^β^) plotted against (1 – *f*) and δ^15^N^SP^ plotted against (1 – *f*) for the H_2_^18^O enriched assays and controls; Figures S5 and S6 display the Rayleigh plots (where *y* = [−*f *ln *f*/(1 – *f*)]) for δ^18^O, δ^15^N, and δ^15^N^SP^ for the experiments performed to determine KIEs; Scheme S1 shows a proposed modification of the *cis*-heme *b*_3_ mechanism; Tables S1 and S2 present the N_2_O concentrations, *f* values, and δ values for enzymatic N_2_O production; description of the accompanying standard deviation calculations is also included in the Supporting Information; Table S3 includes the ε and KIE values and associated statistics for the average of 1000 bootstrapped samples (PDF).

## Abbreviations

cNOR: cytochrome *c* NOR
δ^15^N^SP^: site preference
DDM: *n*-dodecyl-β-d-maltoside
DFT: density functional theory
DNRA: dissimiliatory nitrate reduction to ammonia
ECD: electron capture detector
EIE: equilibrium isotope effect
EIE_int_: intrinsic EIE
EXC: excited vibrational states
Fe_B_: nonheme iron center in cNOR
Fe_*b*3_: iron center of heme *b*_3_ in cNOR
GC: gas chromatography
HEPES: 4-(2-hydroxyethyl)-1-piperazineethanesulfonic acid
IRMS: isotope ratio mass spectrometer
KIE: kinetic isotope effect
KIE_int_: intrinsic KIE
KIE_obs_: observed KIE
MMI: mass and moments of inertia
NOR: nitric oxide reductase
qNOR: quinol dependent NOR
TMPD: *N,N,N*′*,N*′-tetramethyl-*p*-phenylenediamine
TS: transition state
TSDF: transition state decomposition frequency
ZPE: zero-point energy
