# Supplementary material for: Isotopic Fractionation and Kinetic Isotope Effects of a Purified Bacterial Nitric Oxide Reductase (NOR)
Source: Biochemistry. 2025 Oct 10;64(20):4327–40. doi: 10.1021/acs.biochem.5c00417 (PMC12548093; doi:10.1021/acs.biochem.5c00417)
Supplement: Supplementary file 1 [file bi5c00417_si_001.pdf]

Supporting Information

for

Isotopic Fractionation and Kinetic Isotope Effects of a Purified Bacterial Nitric Oxide Reductase (NOR)

Elise D. Rivett[†,○], *Clarisse M. Finders*[†,○], *Joshua A. Haslun*[†,○], *Hasand Gandhi*[‡,○], *Maximilian Kahle*[§], *Pia Ädelroth*[§], *Peggy H. Ostrom*[‡,○], *Nathaniel E. Ostrom*[‡,○,*], *Eric L. Hegg*[†,○,*]

[†]Department of Biochemistry & Molecular Biology, Michigan State University, East Lansing, Michigan 48824, United States

[○]DOE Great Lakes Bioenergy Research Center, Michigan State University, East Lansing, MI, 48824, United States

[‡]Department of Integrative Biology, Michigan State University, East Lansing, Michigan 48824, United States

[§]Department of Biochemistry and Biophysics, Stockholm University, SE-106 91 Stockholm, Sweden

***Correspondence to:**

Eric L. Hegg, 313A Biochemistry, Michigan State University, East Lansing, MI 48824-1319.

Phone: (517) 353-7120; E-mail: erichegg@msu.edu

Nathaniel E. Ostrom, E-mail: ostromn@msu.edu

Standard deviation calculations for N_2O produced, initial NO, and *f* values

The fraction of substrate remaining, *f*, is calculated using Eq. S1

$$f = \frac{N_s}{N_{s0}} = 1 - \frac{2 * n_{N2O,total}}{n_{NO,initial}} \quad \text{(S1)}$$

where $n_{N2O,total}$ is the total amount of N_2O produced at that timepoint (in μmol) and $n_{NO,\ initial}$ is the amount of NO initially added (10.3 μmol, calculated using the ideal gas law). Determining the standard deviation for each *f* value requires calculating the standard deviation for $n_{N2O,total}$ and $n_{NO,\ initial}$.

The total amount of N_2O produced, $n_{N2O,total}$, is calculated by adding the amount of N_2O remaining in the reaction bottle and the amount of N_2O removed during previous sampling:

$$n_{N2O,total} = [n_{N2O,rxn\ bottle}] + [n_{N2O,previous\ samples}] \quad \text{(S2)}$$

The amount of N_2O in the reaction bottle is determined by multiplying the concentration of the N_2O in the current sample (in μmol/mL) by the total volume of the reaction bottle headspace (147.2 ± 0.6 mL) (Eq. S3). To determine sample concentration, the amount of N_2O in the sample ($n_{N2Osample}$, determined using a standard curve) is divided by the sample volume (V_{sample}).

$$n_{N2O,total} = \frac{n_{N2O,sample}}{V_{sample}} * V_{headspace} + [n_{N2O,previous\ samples}] \quad \text{(S3)}$$

Thus, determining the standard deviation for $n_{N2O,total}$ ($\sigma_{nN2O,total}$) requires combining the standard deviations for each term listed in Eq. S3 via error propagation.[1] The standard deviation for each $n_{N2Osample}$ value ($\sigma_{nN2O,sample}$) was calculated according to the method previously described for finding standard deviations for individual sample concentrations determined using a calibration curve.[2, 3] The standard deviation for V_{sample} ($\sigma_{Vsample}$) was estimated to be 0.01* V_{sample} based on the accuracy of the gastight syringe used to sample the headspace. The standard deviation for $V_{headspace}$ ($\sigma_{Vheadspace}$) was 0.6 mL, based on 10 measurements of the total volume of the 160 mL serum bottles used as the reaction bottle. Due to the small amount of N_2O removed during each timepoint relative to the total amount of N_2O in the reaction bottle, the standard deviation for N_2O removed during previous sampling timepoints was negligible and was omitted. The overall equation for the standard deviation for $n_{N2O,total}$ is written as follows (Eq. S4):

$$\sigma_{n_{N2O,total}} = [n_{N2O,total}] * \sqrt{\left(\frac{\sigma_{n_{N2O,sample}}}{n_{N2O,sample}}\right)^2 + \left(\frac{\sigma_{V\ sample}}{V\ sample}\right)^2 + \left(\frac{\sigma_{V_{headspace}}}{V_{headspace}}\right)^2} \quad \text{(S4)}$$

Standard deviations for $n_{N2O,total}$ ranged from 0.03 to 0.21 μmol (Tables S1-S2).

The standard deviation for the amount of NO initially added ($\sigma_{nNO,\ initial}$) was calculated using the following values for the volume (*V*) and temperature (*T*) terms in the ideal gas law: $V_{NO} \pm \sigma_{VNO}$ = 0.00025 ± 0.01*(0.00025) L; $T \pm \sigma_T$ = 296 ± 1 K (Eq. S5).

$$\sigma_{n_{NO,initial}}(\mu mol) = \left[n_{NO,initial}(mol)\right] * \sqrt{\left(\frac{\sigma_{V_{NO}}}{V_{NO}}\right)^2 + \left(\frac{\sigma_T}{T}\right)^2} * 10^6 \, {}^{\mu mol}/_{mol} \quad \text{(S5)}$$

Using Eq. S5, $\sigma_{nNO,\ initial}$ was estimated to be 0.11 μmol.

Finally, $\sigma_{nN2O,total}$ and $\sigma_{nNO,\ initial}$ were combined to determine the standard deviation for each *f* value (σ_f) (Eq. S6).

$$\sigma_f = f * \sqrt{\left(\frac{\sigma_{n_{N2O,total}}}{n_{N2O,total}}\right)^2 + \left(\frac{\sigma_{n_{NO,initial}}}{n_{NO,initial}}\right)^2} \quad \text{(S6)}$$

The values of σ_f ranged from 0.01-0.06 (Tables S1-S2).

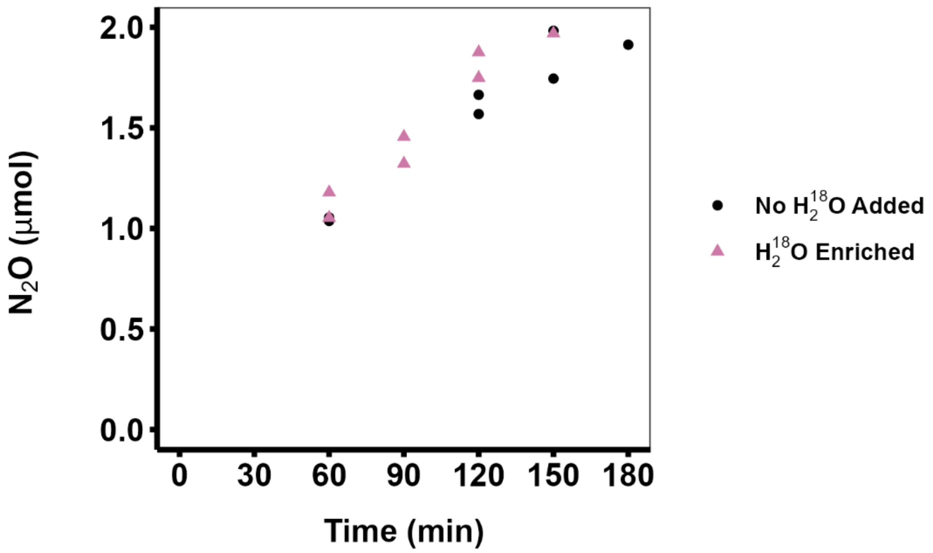

Figure S1. Production of N_2O by *P. denitrificans* cNOR with ^{18}O enriched water. The data for two replicates with ^{18}O enriched water (87‰) is shown as purple triangles, and the data for two replicates without ^{18}O enriched water is shown as black circles. Each replicate contained 78 nM cNOR and 10.3 µmols of NO gas, and the maximum amount of N_2O that could be produced was therefore 5.15 µmols.

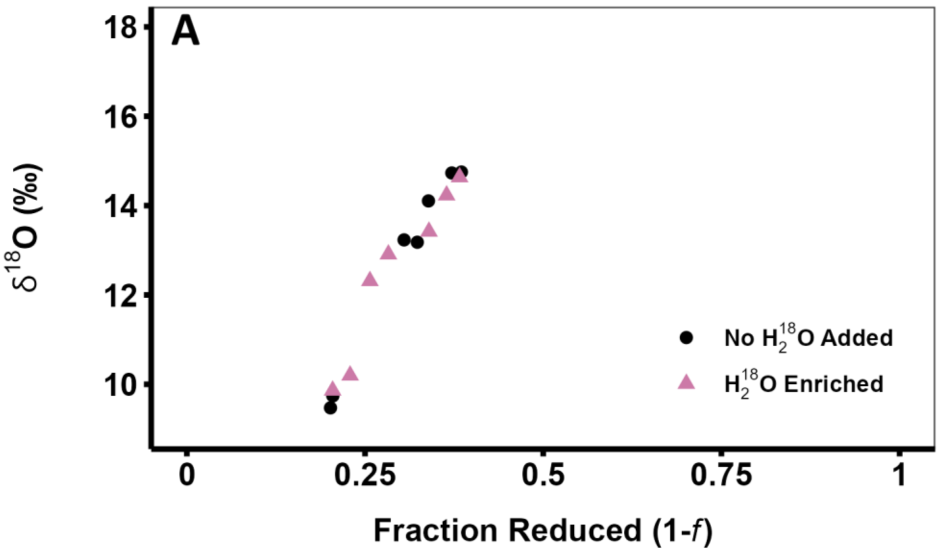

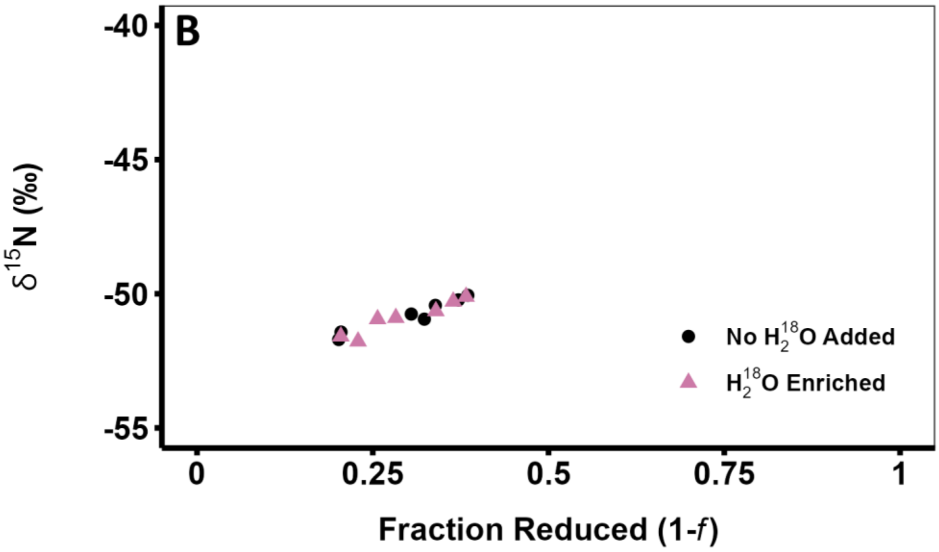

Figure S2. $\delta^{18}O$ (A) and $\delta^{15}N$ (B) of N_2O produced by cNOR as a function of the fraction of substrate (NO) reduced (1-*f*) with ^{18}O enriched water (purple triangles) and unenriched water (black circles). The reaction progresses from left to right. For each condition (enriched and unenriched water), the data from two replicates were combined.

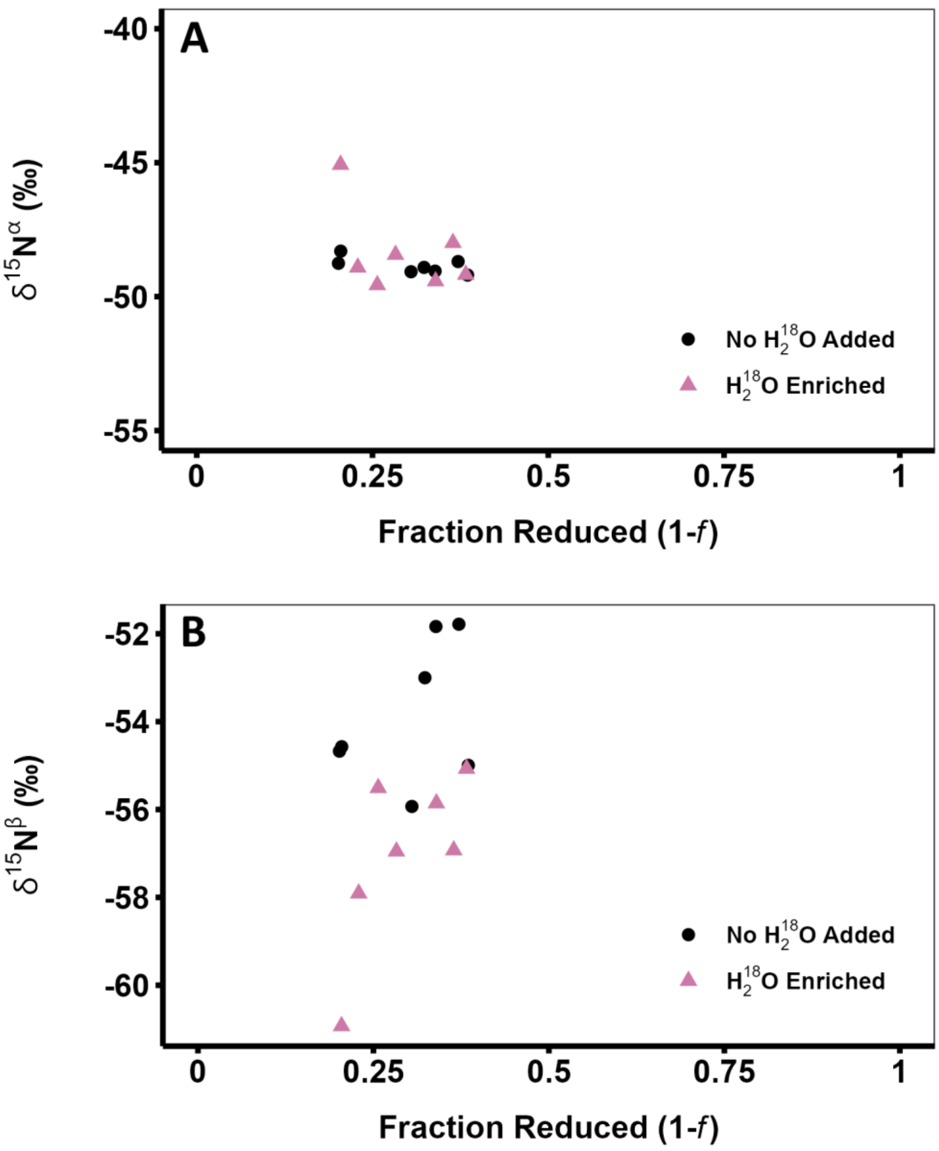

Figure S3. $\delta^{15}N^{\alpha}$ (A) and $\delta^{15}N^{\beta}$ (B) of N_2O produced by cNOR as a function of the fraction of substrate (NO) reduced (1-*f*) with the reaction medium containing either ^{18}O enriched water (purple triangles) or unenriched water (black circles). The reaction progresses from left to right. For each condition (enriched and unenriched water), the data from two replicates were combined.

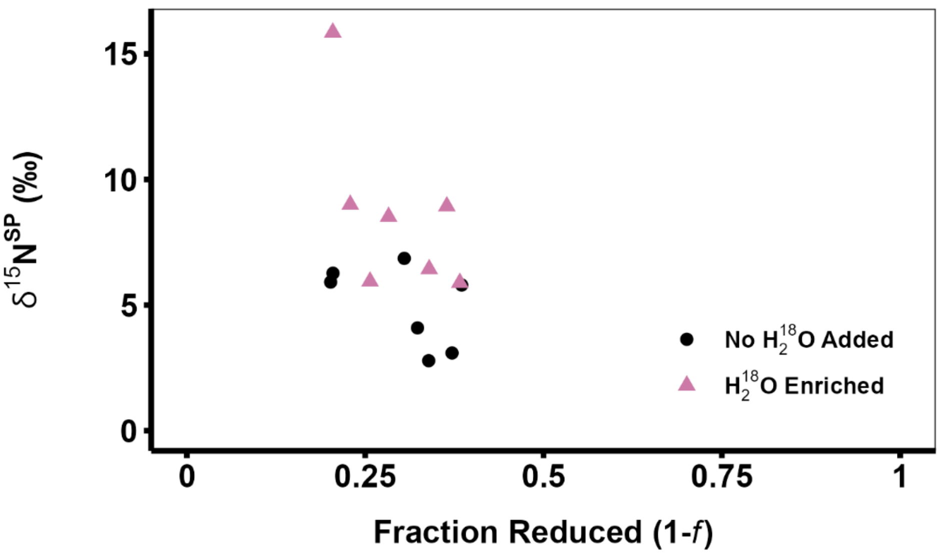

Figure S4. $\delta^{15}N^{SP}$ ($\delta^{15}N^{SP} = \delta^{15}N^{\alpha} - \delta^{15}N^{\beta}$) of N_2O produced by cNOR as a function of the fraction of substrate (NO) reduced (1-*f*) with the reaction medium containing either ^{18}O enriched water (purple triangles) or unenriched water (black circles). The reaction progresses from left to right. For each condition (enriched and unenriched water), the data from two replicates were combined.

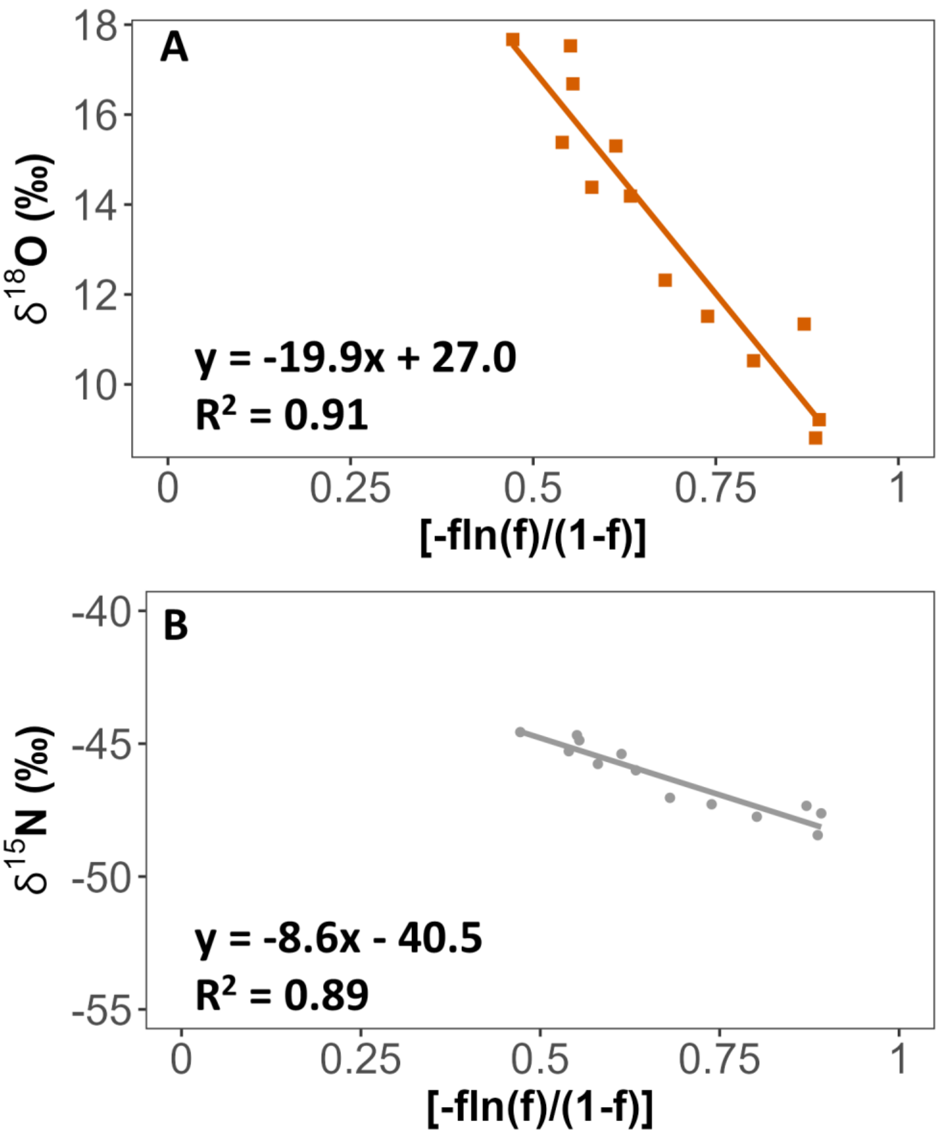

Figure S5. Standard Rayleigh plot for $\delta^{18}O$ (A) and $\delta^{15}N$ (B) of N_2O produced by cNOR plotted as a function of [-*f*ln*f*/(1-*f*)] with the reaction progressing from right to left. The $\delta^{18}O$ values are shown as orange squares, and the $\delta^{15}N$ values are shown as gray circles. A linear fit of the data to Mariotti's approximation of the Rayleigh equation[4] (Eq. 4, main text) is shown as a solid line in each plot. Note that the slope of the plot in panel A does not represent the true enrichment factor (ε)[4] for $\delta^{18}O$ (*i.e.*, $\varepsilon = (R_{pi}/R_s - 1)*1000$) because the $\delta^{18}O$ values for the O atom that went to H_2O were not measured. The data from three biological replicates were pooled.

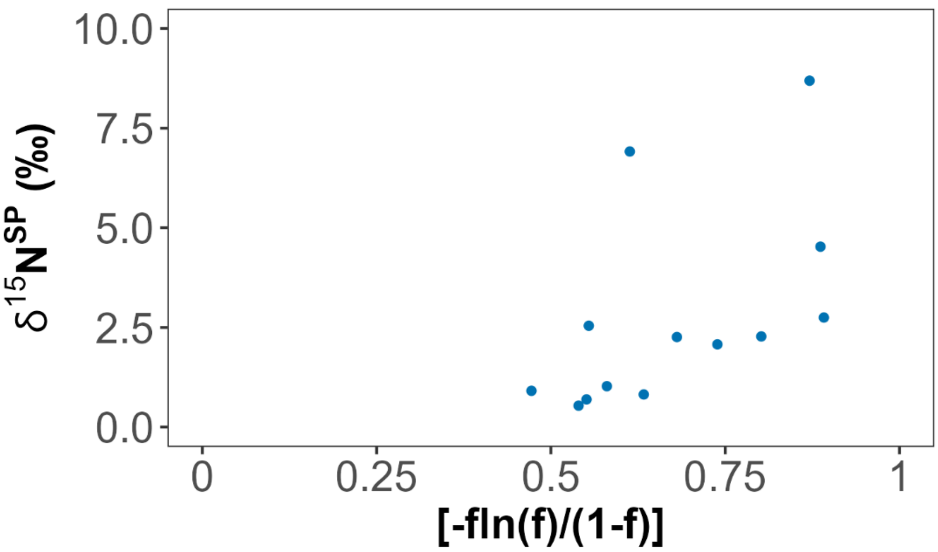

Figure S6. Standard Rayleigh plot for site preference $\delta^{15}N^{SP}$ ($\delta^{15}N^{SP} = \delta^{15}N^{\alpha} - \delta^{15}N^{\beta}$) of N_2O produced by cNOR plotted as a function of [-*f*ln*f*/(1-*f*)] with the reaction progressing from right to left. The $\delta^{15}N^{SP}$ values are shown as blue circles. The data fit poorly to Mariotti's approximation of the Rayleigh equation[4] (Eq. 4, main text) ($R^2 = 0.29$), indicating that there is no significant trend in $\delta^{15}N^{SP}$ over the course of the reaction.

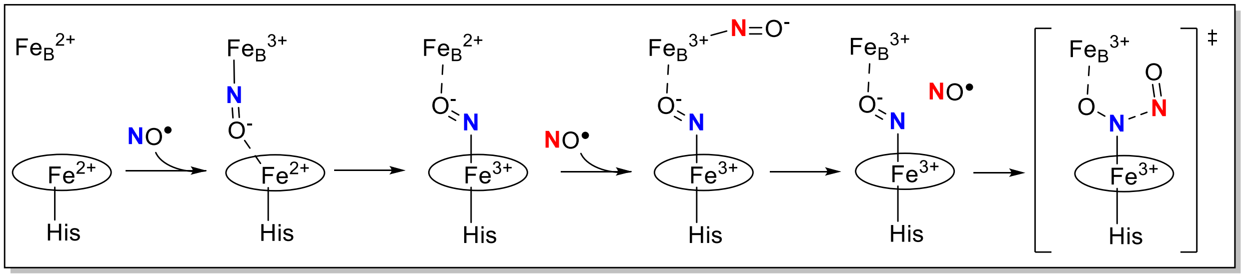

Scheme S1. Potential modified *cis*-heme b_3 mechanism for cNOR showing pre-equilibrium binding steps where the first and second NO transiently bind Fe_B(II). All Fe-nitrosyl ($\{FeNO\}^7$) complexes are shown as Fe(III)-NO^- as this formal oxidation state is likely the best description of each of these complexes (see main text Discussion). Previously published time-resolved spectroscopic data suggests that the first NO (blue) rapidly binds Fe_B(II) before migrating to heme b_3.[5, 6] In the modified heme b_3 mechanism, the second NO (red) then transiently binds Fe_B(II), although not necessarily in a configuration amenable to producing a *trans*-hyponitrite intermediate. (DFT calculations suggest that a dinitrosyl intermediate with NO bound to Fe_B in an equatorial position is more favorable than positioning the nitrosyl groups closer together.)[7] The second NO can then dissociate from Fe_B (effectively producing a caged radical) before attacking the $\{Fe_{b3}\text{-}NO\}^7$ complex to produce the same transition state proposed for the original *cis*-heme b_3 mechanism. The rest of the mechanism then proceeds as previously proposed by Blomberg[8] (main text Scheme 3). By adding a pre-equilibrium binding step for the second NO molecule, this modified mechanism provides one possible explanation for our observation that both N atoms in N_2O have normal KIEs.

Table S1. N_2O concentrations and δ values for N_2O produced from NO by purified *P. denitrificans* cNOR.

**Replicate**	**Time (min)**	**N_2O (µmol)** [a]	**f** [a]	**$\delta^{18}O$ (‰)** [b]	**$\delta^{15}N$ (‰)** [b]	**$\delta^{15}N^{\alpha}$ (‰)** [c]	**$\delta^{15}N^{\beta}$ (‰)** [c]	**$\delta^{15}N^{SP}$ (‰)** [d]
1	40	1.22 ± 0.09	0.76 ± 0.06	11.3	-47.3	-43	-51.7	8.7
1	120	3.07 ± 0.16	0.40 ± 0.02	15.3	-45.4	-41.9	-48.9	6.9
1	160	3.4 ± 0.18	0.34 ± 0.02	16.7	-44.9	-43.6	-46.2	2.5
1	200	3.42 ± 0.19	0.34 ± 0.02	17.5	-44.7	-44.3	-45	0.7
1	240	3.81 ± 0.21	0.26 ± 0.01	17.7	-44.6	-44.1	-45	0.9
2	40	1.08 ± 0.06	0.79 ± 0.04	8.8	-48.4	-45.5	-50	4.5
2	80	2.26 ± 0.07	0.56 ± 0.02	11.5	-47.3	-46.4	-48.5	2.1
2	120	3.26 ± 0.09	0.37 ± 0.01	14.4	-45.8	-45.8	-46.8	1
3	40	1.04 ± 0.06	0.80 ± 0.04	9.2	-47.6	-45.5	-48.3	2.7
3	80	1.79 ± 0.07	0.65 ± 0.03	10.5	-47.8	-46.3	-48.6	2.3
3	120	2.66 ± 0.08	0.48 ± 0.02	12.3	-47	-46	-48.2	2.3
3	160	2.95 ± 0.09	0.43 ± 0.01	14.2	-46	-45.6	-46.4	0.8
3	200	3.48 ± 0.11	0.32 ± 0.01	15.4	-45.3	-45.1	-45.6	0.5

[a] Value ± standard deviation. Standard deviation values were calculated using a standard curve for N_2O concentration and appropriate error propagation techniques. (See "Standard deviation calculations for N_2O produced, initial NO, and *f* values" section above.)

[b] The standard deviation for $\delta^{18}O$ and $\delta^{15}N$ values is 0.5‰.

[c] The standard deviation for $\delta^{15}N^{\alpha}$ and $\delta^{15}N^{\beta}$ values is 0.7‰.

[d] The standard deviation for $\delta^{15}N^{SP}$ is 1.2‰.

Table S2. N_2O concentrations and δ values for N_2O produced from NO by purified *P. denitrificans* cNOR in unenriched water or ^{18}O enriched water.

Replicate	Time (min)	N_2O (μmol) [a]	*f* [a]	$\delta^{18}O$ (‰) [b]	$\delta^{15}N$ (‰) [b]	$\delta^{15}N^{\alpha}$ (‰) [c]	$\delta^{15}N^{\beta}$ (‰) [c]	$\delta^{15}N^{SP}$ (‰) [d]
Unenriched water 1	60	1.04 ± 0.06	0.8 ± 0.05	9.5	-51.7	-48.8	-54.7	5.9
Unenriched water 1	120	1.66 ± 0.07	0.68 ± 0.03	13.2	-50.9	-48.9	-53	4.1
Unenriched water 1	150	1.74 ± 0.08	0.66 ± 0.03	14.1	-50.4	-49	-51.8	2.8
Unenriched water 1	180	1.91 ± 0.09	0.63 ± 0.03	14.7	-50.2	-48.7	-51.8	3.1
Unenriched water 2	60	1.18 ± 0.03	0.77 ± 0.02	10.2	-51.8	-48.9	-57.9	9
Unenriched water 2	90	1.46 ± 0.04	0.72 ± 0.02	12.9	-50.9	-48.4	-57	8.5
Unenriched water 2	120	1.88 ± 0.05	0.64 ± 0.02	14.2	-50.3	-48	-56.9	8.9
^{18}O enriched water 1	60	1.05 ± 0.06	0.8 ± 0.05	9.7	-51.4	-48.3	-54.6	6.3
^{18}O enriched water 1	120	1.57 ± 0.04	0.7 ± 0.02	13.2	-50.8	-49.1	-55.9	6.9
^{118}O enriched water 1	150	1.98 ± 0.05	0.61 ± 0.02	14.8	-50.1	-49.2	-55	5.8
^{18}O enriched water 2	60	1.05 ± 0.03	0.8 ± 0.03	9.9	-51.6	-45.1	-60.9	15.9
^{18}O enriched water 2	90	1.32 ± 0.04	0.74 ± 0.02	12.3	-50.9	-49.6	-55.5	5.9
^{18}O enriched water 2	120	1.75 ± 0.05	0.66 ± 0.02	13.4	-50.6	-49.4	-55.9	6.4
^{18}O enriched water 2	150	1.97 ± 0.05	0.62 ± 0.02	14.6	-50.1	-49.2	-55.1	5.9

[a] Value ± standard deviation. Standard deviation values were calculated using a standard curve for N_2O concentration and appropriate error propagation techniques. (See "Standard deviation calculations for N_2O produced, initial NO, and *f* values" section above.)

[b] The standard deviation for $\delta^{18}O$ and $\delta^{15}N$ values is 0.5‰.

[c] The standard deviation for $\delta^{15}N^{\alpha}$ and $\delta^{15}N^{\beta}$ values is 0.7‰.

[d] The standard deviation for $\delta^{15}N^{SP}$ is 1.2‰.

Table S3. Calculated isotopic enrichment factors (ε), kinetic isotope effects (KIEs), and associated statistics averaged from 1000 bootstrapped samples[a] for N of N_2O produced by purified *P. denitrificans* cNOR.

**Measurement**	**ε (‰)**	**KIE**	**R^2 [b]**	**Linear RMSE [b]**	**Nonlinear RMSE [c]**
^{15}N	-8.7 ± 1	1.0087 ± 0.0010	0.89 ± 0.04	0.38 ± 0.06	NA
$^{15}N^{\alpha}$	-7.3 ± 1.1	1.0073 ± 0.0012	0.89 ± 0.04	0.38 ± 0.06	0.54 ± 0.17
$^{15}N^{\beta}$	-10.0 ± 0.9	1.0101 ± 0.0009	0.89 ± 0.04	0.38 ± 0.06	0.54 ± 0.17

[a] Each bootstrapped data set was generated by randomly sampling the combined experimental data from three replicates (13 observations) 13 times with replacement.

[b] Average value ± standard deviation (1000 bootstrapped samples) for linear regression of bulk $\delta^{15}N$ (dependent variable) against $[-f\ln f/(1-f)]$ (Eq. 4, main text). $R^2 = [1 - (SSR/SST)]$, where *SSR* is the residual sum of squares and *SST* is the total sum of squares. RMSE = root mean square error = $\sqrt{(SSR/n)}$.

[c] Average RMSE value ± standard deviation (1000 bootstrapped samples) for nonlinear regression (Eq. 10, main text) where bulk $\delta^{15}N$ is the dependent variable.

REFERENCES

[1] Caldwell, J.; Vahidsafa, A. *Propagation of Error.* https://chem.libretexts.org/@go/page/353 (accessed 2025-02-20).
[2] Haas, K. 2.5: *Uncertainty in Values Determined from a Calibration Curve.* https://chem.libretexts.org/@go/page/407082 (accessed 2025-02-13).
[3] Miller, J. N. (1991) Basic statistical methods for analytical chemistry. Part 2. Calibration and regression methods. A review, *Analyst 116*, 3-14.
[4] Mariotti, A., Germon, J. C., Hubert, P., Kaiser, P., Letolle, R., Tardieux, A., and Tardieux, P. (1981) Experimental determination of nitrogen kinetic isotope fractionation: Some principles; illustration for the denitrification and nitrification processes, *Plant Soil 62*, 413-430.
[5] Takeda, H., Kimura, T., Nomura, T., Horitani, M., Yokota, A., Matsubayashi, A., Ishii, S., Shiro, Y., Kubo, M., and Tosha, T. (2020) Timing of NO binding and protonation in the catalytic reaction of bacterial nitric oxide reductase as established by time-resolved spectroscopy, *Bull Chem Soc Jpn 93*, 825-833.
[6] Takeda, H., Shimba, K., Horitani, M., Kimura, T., Nomura, T., Kubo, M., Shiro, Y., and Tosha, T. (2023) Trapping of a mononitrosyl nonheme intermediate of nitric oxide reductase by cryo-photolysis of caged nitric oxide, *J Phys Chem B 127*, 846-854.
[7] Blomberg, M. R. A. (2017) Can reduction of NO to N_2O in cytochrome *c* dependent nitric oxide reductase proceed through a *trans*-mechanism?, *Biochemistry 56*, 120-131.
[8] Blomberg, M. R. A. (2021) The importance of exact exchange-A methodological investigation of NO reduction in heme-copper oxidases, *J Chem Phys 154*, 055103.
